# A novel role of TRIM28 B box domain in L1 retrotransposition and ORF2p-mediated cDNA synthesis

**DOI:** 10.1093/nar/gkad247

**Published:** 2023-04-18

**Authors:** Qianhui Du, Emily C Stow, Dawn LaCoste, Benjamin Freeman, Melody Baddoo, Afzaal M Shareef, Kyle M Miller, Victoria P Belancio

**Affiliations:** Tulane Cancer Center, Tulane Health Sciences Center, 1700 Tulane Ave, New Orleans, LA 70112, USA; Department of Structural and Cellular Biology, Tulane School of Medicine, 1430 Tulane Ave, New Orleans 70112, USA; Tulane Cancer Center, Tulane Health Sciences Center, 1700 Tulane Ave, New Orleans, LA 70112, USA; Department of Structural and Cellular Biology, Tulane School of Medicine, 1430 Tulane Ave, New Orleans 70112, USA; Tulane Cancer Center, Tulane Health Sciences Center, 1700 Tulane Ave, New Orleans, LA 70112, USA; Department of Structural and Cellular Biology, Tulane School of Medicine, 1430 Tulane Ave, New Orleans 70112, USA; Tulane Cancer Center, Tulane Health Sciences Center, 1700 Tulane Ave, New Orleans, LA 70112, USA; Department of Structural and Cellular Biology, Tulane School of Medicine, 1430 Tulane Ave, New Orleans 70112, USA; Tulane Cancer Center, Tulane Health Sciences Center, 1700 Tulane Ave, New Orleans, LA 70112, USA; Tulane Cancer Center, Tulane Health Sciences Center, 1700 Tulane Ave, New Orleans, LA 70112, USA; Department of Structural and Cellular Biology, Tulane School of Medicine, 1430 Tulane Ave, New Orleans 70112, USA; Department of Molecular Biosciences, Institute for Cellular and Molecular Biology, University of Texas at Austin, 100 E 24th Street, Austin, TX 78712, USA; Tulane Cancer Center, Tulane Health Sciences Center, 1700 Tulane Ave, New Orleans, LA 70112, USA; Department of Structural and Cellular Biology, Tulane School of Medicine, 1430 Tulane Ave, New Orleans 70112, USA

## Abstract

The long interspersed element 1 (LINE-1 or L1) integration is affected by many cellular factors through various mechanisms. Some of these factors are required for L1 amplification, while others either suppress or enhance specific steps during L1 propagation. Previously, TRIM28 has been identified to suppress transposable elements, including L1 expression via its canonical role in chromatin remodeling. Here, we report that TRIM28 through its B box domain increases L1 retrotransposition and facilitates shorter cDNA and L1 insert generation in cultured cells. Consistent with the latter, we observe that tumor specific L1 inserts are shorter in endometrial, ovarian, and prostate tumors with higher TRIM28 mRNA expression than in those with lower TRIM28 expression. We determine that three amino acids in the B box domain that are involved in TRIM28 multimerization are critical for its effect on both L1 retrotransposition and cDNA synthesis. We provide evidence that B boxes from the other two members in the Class VI TRIM proteins, TRIM24 and TRIM33, also increase L1 retrotransposition. Our findings could lead to a better understanding of the host/L1 evolutionary arms race in the germline and their interplay during tumorigenesis.

## INTRODUCTION

The only known autonomously active transposable element (TE) in humans, long interspersed element 1 (LINE-1 or L1), belongs to the class of non-long terminal repeat (non-LTR) retroelements (1–4). The L1 sequence is composed of a 5’ untranslated region (UTR) containing a Pol II promoter, two open reading frames (ORFs), a 3’UTR, and a poly(A) signal (5–7). ORF1 protein (ORF1p) is the main structural protein of the L1 ribonucleoprotein particle (RNP), a necessary intermediate in the L1 replication cycle (8–13). ORF2 protein (ORF2p) harbors the endonuclease and reverse transcriptase activities required for retrotransposition of both L1 and its parasite Alu (14–16). After gaining access to nuclear chromatin, ORF2p endonuclease cleaves genomic DNA (15,17–20). The resulting DNA break is used by the ORF2p reverse transcriptase for a process termed target primed reverse transcription (TPRT) (15,21,22). During TPRT ORF2p reverse transcribes its associated L1mRNA into a cDNA using single-stranded genomic DNA as a primer. Aided by cellular factors this ultimately leads to the integration of a new L1 copy into the host genome (21,23,24). Most of these *de novo* inserts are 5’ truncated and therefore are incapable of further amplification (25–27). Although the exact mechanism leading to truncated integration events is unknown, it is speculated that cellular DNA repair, unknown host factors, and/or limited L1 RT processivity could be involved (28–33). As is true with many multifunctional proteins, ORF2p endonuclease and reverse transcriptase domains remain functional even when separated from each other (34). Specific truncated EN- and RT-containing fragments can reconstitute Alu retrotransposition when supplied *in trans* (34).

Retrotransposition events were initially detected in the germline, and during early embryogenesis (35). Some of these events are mutagenic causing heritable diseases, such as hemophilia, or neurofibromatosis (36). L1 expression and retrotransposition are also well documented in a variety of human epithelial cancers (37–39). More recently, it became evident that L1 expression and retrotransposition also occur in normal adult cells (25,40–43). To maintain genome integrity and avoid accumulation of genetic defects, mammalian cells have developed various mechanisms to suppress retrotransposition (44–47). These mechanisms include epigenetic silencing of the L1 promoter, RNA interference, and numerous cellular proteins involved in DNA damage responses (47). In contrast, host factors enhancing L1 retrotransposition are less abundant and those that play a pleotropic role in L1 retrotransposition are rare.

Tripartite motif-containing (TRIM) family proteins are crucial members in the innate immune response and have a broad effect on restricting viruses and retroelements (48,49). It has been reported that human TRIM5α contributes to safeguarding the genome by downregulating L1 retrotransposition (49). Studies have also shown that TRIM28 depletion resulted in upregulation of a range of retroelements, especially the endogenous retroviruses (ERVs), due to the loss of H3K9me3 repressive chromatin mark (50–53). It is reported that TRIM28 facilitates L1 silencing in mouse fibroblasts by establishing transcriptionally repressive heterochromatin upon mono-ADP ribosylation by SIRT6 protein (54). ChIP-Seq studies identified TRIM28 binding sites in the 5’UTRs of older L1 subfamilies (55). These sequences can suppress reporter gene expression in a cell type specific manner (56). They may also suppress L1 expression, although some studies used RT-qPCR to measure L1 expression, which does not provide accurate results (54,55). Other studies reported that KZNF genes cooperated with TRIM28 to suppress primate specific L1s during their expansion, with younger L1 subfamilies escaping this suppression by purging KZNF binding site (57). In general, current literature supports that, compared to ERVs, endogenous L1 expression appears to be less affected by TRIM28 (52,53) and that, at the level of transcriptional regulation, TRIM28 only suppresses certain mid-aged L1 subfamilies, not L1Hs, at least in stem cells (55). In addition to its involvement in silencing of L1 expression, a study using genome-wide CRISPR-Cas9 screen in human chronic myeloid leukemia K562 cells using an L1-G418R retrotransposition reporter identified TRIM28 as a negative regulator by comparing the frequencies of sgRNAs with deep sequencing and analysis using Cas9 high-throughput maximum likelihood estimator (CasTLE) (58). On the other hand, TRIM28 has been identified as a positive regulator of L1 activity in a genome-wide CRISPR/Cas9 screen with HeLa M2 cells using an L1-G418R reporter, where the negative and positive L1 regulators were determined by the percentage of GFP positive cells (32). Thus, understanding the involvement of TRIM28 in L1 replication cycle would establish this protein as a cellular factor with potential pleotropic roles in L1 retrotransposition.

TRIM28 gene, also known as KAP1 (Krüppel-Associated Box (KRAB)-Associated Protein 1) or TIF1-β (Transcriptional Intermediary Factor 1 β), encodes for TRIM28 protein, a member of the Class VI TRIM family proteins, which share many structural characteristics (59–63). TRIM28 protein, like the other two members in the class VI TRIM proteins, TRIM24 and TRIM33, is composed of multiple conserved structural domains including a RING (Really Interesting New Gene) finger, two B boxes (B1 and B2), and a leucine zipper coiled-coil region (CC) in the N-terminus. These domains are collectively called the RBCC or TRIM domain (64,65). TRIM28 also contains N-CoR2 Homology Domain (NHD), the plant homeodomain (PHD) finger and a Bromodomain in the C-terminus (65–68). The RBCC domain is responsible for the interaction with the KRAB domain present in a very large set of the KRAB-Zinc Finger (KRAB-ZFP) transcription factors (59,66,69). The PHD domain of TRIM28 can act as an intramolecular SUMO E3 ligase, SUMOylating several lysines in the bromodomain (68). KAP1 SUMOylation is required for recruitment of SETDB1 and NuRD, which are critical factors in creation of H3K9me3 marks and deacetylation of histone proteins, leading to formation of condensed heterochromatin required for KRAB- TRIM28-mediated gene repression (68,70). Recently, the biophysical and structural approaches revealed that TRIM28 forms antiparallel dimers via its coil-coil domain (56). These TRIM28 dimers assemble into tetramers and higher-order oligomers in a concentration-dependent manner through the B box domain (56,71). It is reported that the higher-order oligomers are not required for TRIM28 function in transcriptional silencing (56). Higher TRIM28 gene expression has been linked to a more aggressive disease in several types of cancers and is associated with poor prognosis (72–76), suggesting that TRIM28 oligomerization could be contributing to tumorigenesis. All three members of the class VI TRIM proteins share high-level conserved amino acid sequences in their B box domain (69) and have been shown to be aberrantly expressed in multiple cancer types (77).

In this study using previously reported ORF2p fragments (18,34,78) and TRIM28 fragments (56,68), we show that TRIM28 increases L1 retrotransposition in its oligomer-forming B box domain-dependent manner. TRIM28 B box as well as B boxes from TRIM24 and TRIM33 are sufficient to increase L1 retrotransposition. We also discover a novel role of TRIM28 and its B box in the ORF2p-mediated cDNA synthesis, which is supported by measuring the length of cDNA products and *de novo* L1 inserts in transiently transfected cultured cells and by analysis of *de novo* tumor-specific L1 inserts in tumors of patients with endometrial cancer, prostate cancer, and ovarian cancer. Together, our studies have identified previously unknown effects of TRIM28 on potentially distinct steps in L1 retrotransposition cycle and provided patient-based data supporting *in vivo* relevance of our findings.

## MATERIALS AND METHODS

### Plasmids

Plasmids containing DNA encoding human and mouse TRIM28 were obtained from Origene (RC201205 and MR210883, respectively) and subcloned into a PCDNA 3.1/Hygro+ vector using NheI and HindIII restriction enzymes. The coding sequences for fragments of human TRIM28 were synthesized by Genescript, including RBCCH (AA 1–509), RBB (AA 1–244), RING (AA 1–139), BB (AA 139–244), CC (AA 244–405), N-P-Br (AA 509–835), N-P (AA 509–680), P-Br (AA 617–835), NHD (AA 509–617), PHD (AA 617–680), Br (AA 680–835). The break points for TRIM28 domains were used as described (56,68). The mutant TRIM28 constructs were also generated by Genescipt based on (56), including BB A160D, BB T163A, BB E175R, BB 3M and TRIM28 3M. The human TRIM28 DNA sequence was used as reference. All TRIM28 constructs were tagged with FLAG at the C terminus and cloned into a PCDNA 3.1/Hygro+ expression plasmid.

The ORF2 constructs were generated as previously described (18,34,78). Specifically, Cryptic-T7 (ORF2 AA 247–380) was tagged with T7 on the C-terminus. T7-EN (ORF2 AA 1–239) T7-ZRT (ORF2 AA 380–773), T7-Z (ORF2 AA 380–480), T7-RT (ORF2 AA 511–773), T7-Cterm (ORF2 AA 774–1275) were all tagged with T7 on the N-terminus. All ORF2 fragments were cloned in the PCDNA3.1/Hygro + expression plasmid.

### Cell lines

HeLa cells were maintained as previously described (79). U2OS cells were maintained in Dulbecco's modified Eagle's medium high glucose (GIBCO), 10% fetal bovine serum.

The U2OS TRIM28 KO cell line was generated using CRISPR-Cas9 gene-editing using the protocol as previously described (80). Briefly, pSPCas9(BB)-2A-Puro (pX459, Addgene plasmid ID: 48139) containing the sgRNA sequence CATGCGTGATAGTGGCAGCA targeting TRIM28 was constructed and used for gene targeting. U2OS cells were transiently transfected with this plasmid and subjected to limited selection with PURO to enrich for plasmid containing cells. Cells were then diluted into single colonies in 96 well plates and individual colonies were screened for TRIM28 KO using western blot analysis of endogenous TRIM28 expression, which was preformed using TIF1β #4123 (Cell signaling) Rabbit antibody.

### L1 retrotransposition assay

500 000 cells were seeded 16–18 h prior to transfection in T75 flasks. 0.2 ug L1Neo or 0.6ug L1 Blast expression plasmid (81) and 0.8 ug of other indicated constructs were co-transfected using 12 ul of Plus reagent (Life Technologies) in 180 ul serum free media. Then 85 ul of serum free media was mixed with 15 ul Lipofectamine reagent (Life Technologies) before being added into the previous DNA mix. The transfection cocktail was added to the cells in 6ml of serum free media for 3 h, after which it was replaced with 9 ml of minimum essential media (MEM). Approximately 24 h later the media was replaced with 10 ml of selective medium containing 500 μg/ml G418 (Life Technologies) or 10 μg/ml of Blasticidin (InvivoGen) for 14 days. Selective medium was changed every 3 days. The cells were then fixed and stained with crystal violet solution (0.2% crystal violet in 5% acetic acid and 2.5% isopropanol). The number of Neomycin/Blastcidin-resistant colonies were counted in each flask. Statistical analysis was performed using unpaired two sample t test compared with control.

For the L1 retrotransposition assay in Figure 6, 500 000 HeLa cells were seeded 16–18 h prior to transfection in T75 flasks. 0.2 ug L1Neo expression plasmid and 1.2 ug of indicated constructs were used. The rest of transfection steps were the same as described above.

### L1 toxicity assay

Five hundred thousand HeLa cells or U2OS cells were seeded in T75 flasks. Cells were transfected next day using Lipofectamine reagent (Life Technologies) following the manufacturer's protocol with 0.1 μg of pIRES2-EGFP and 0.8ug of indicated constructs. The plasmids were mixed with 12 ul of Plus reagent (Life Technologies) in 180 ul serum free media. Then 85 ul of serum free media was mixed with 15 ul Lipofectamine reagent (Life Technologies) before being added into the previous DNA mix. The transfection cocktail was added to the cells in 6 ml of serum free media for 3 h, after which it was replaced with 9 ml of Minimum Essential Media (MEM). Cells were maintained under selective medium containing 500μg/ml G418 (Life Technologies) for 14 days starting 48 h post transfection before being fixed and stained with crystal violet solution (0.2% crystal violet in 5% acetic acid and 2.5% isopropanol). Selective medium was changed every 3 days. The number of Neomycin-resistant colonies were counted in each flask. Statistical analysis was performed using unpaired two sample t test compared with control.

### Co-immunoprecipitation

Adapted from (82,83), two million HeLa cells were seeded 16–18 h prior to transfection in T75 flasks and co-transfected with 3 ug of each indicated plasmids. 12 ul of Plus reagent (Life Technologies) in 180 ul serum free media was mixed with 24 ul Lipofectamine reagent and 76 ul serum free media (Life Technologies). The transfection cocktail was added to the cells in 6 ml of serum free media for 3 h, after which it was replaced with 9 ml of MEM. Approximately 24 h post-transfection, cells were washed 1× with phosphate buffered saline (PBS) and then harvested in 500 ul total lysis buffer (TLB: 50 mM Tris, 150 mM NaCl, 10 mM EDTA, 0.5% SDS, 0.5% TritonX, pH 7.2) supplemented with 10 ul/ml of Halt protease inhibitor cocktail, phosphate inhibitor cocktail 2, and phosphate inhibitor cocktail 3 (Sigma). Cell lysates were then sonicated with a Microson XL-2000 sonicator (Misonix) 4× (6 s sonication/10 s rest on ice) and centrifuged at 4°C at 15 000 rpm for 15 min. Protein concentrations of cleared cell lysates were determined by BioRad protein assay (Bradford method). 100 μl of FLAG resin (Anti-Flag M2 Affinity Gel Sigma A2220) was centrifuged at 8200g for 30 s, resin was removed of supernatant and washed twice using 500 μl of TBS (50 mM Tris–HCl,150 mM NaCl, 1 mM EDTA, 1% Triton X-100 v/v, pH  7.4). 40 ug of the protein sample was combined with TBS buffer to make up the total volume of 1 ml and incubated with the prepared FLAG resin overnight at 4°C on a revolving platform. Twenty-four hours later, the mixture was centrifuged at 8200g for 30 s to remove the supernatant on top of the resin, which was then washed three times with 500 μl of TBS. After the washes, the remaining protein was eluted by incubation at 100°C for 3 min in 20 μl of 2× sample buffer (125 mM Tris–HCl, 4% SDS, 20% (v/v) glycerol, pH  6.8). The eluted samples were centrifuged for 30 s at 8200g. The supernatant was used for western blot analysis (the co-IP fraction).

### Co-IP with RNAse treatment

Cell lysates were either treated (+) or not treated (−) with 5μl RNase A (cell signaling for 20 min at 25°C. Co-immunoprecipitation was performed as mentioned above.

### Western blot analysis

Cell lysate containing 40ug of total proteins was boiled in equal volume of Laemmli buffer with 3.4% mercaptoethanol. Samples were fractionated on 4–20% Tris–glycine or 3–8% Tris–acetate gels (Life Technologies) and transferred to nitrocellulose membranes using the iBlot2 system (Life Technologies). The XCell Blot Module was used for wet-transfer module for ORF2p analysis. Membranes were blocked in PBS-Tween (PBS, 0.1% Tween) with 5% blotting-grade blocker (BioRad) and incubated with primary antibodies overnight at 4°C. Targeted proteins were detected with corresponding primary antibodies: hORF1 (custom polyclonal rabbit antibody: TGNSKTQSASPPPK) antibody(82,83); abcam anti-T7 tag ab9115; cell signaling anti-FLAG #2368S; cell signaling TIF1β Antibody #4123; cell signaling GAPDH (14C10) Rabbit mAb #2118. Membranes were washed 3× in PBS–Tween for 5 min before incubation with secondary antibodies. Secondary antibody HRP-mouse anti-rabbit (Santa Cruz: sc2357) or HRP-mouse anti-goat (Santa Cruz: sc2354) was applied for 1 h at room temperature (1:5000 in 3% blotting grade blocker/PBS–Tween). Images were captured using a BioRad Gel Doc XR+ imager. GAPDH was used as loading control.

### Nuclear/cytoplasm fractionation

The processing of nuclear and cytoplasmic fractions was performed as previously described (83,84) Specifically, HeLa cells were washed with 1× PBS (137 mM NaCl (Sigma S9888), 2.7 nM KCl (Sigma P4505), 10 mM Na_2_HPO_4_ (Sigma S3264) and 2 mM KH2PO4 (Sigma P9791), pH  7.4) and harvested using 500 μl of TLB (50 mM Tris, 150 mM NaCl, 10 mM EDTA, TritonX-100 0.5% v/v, Halt Protease inhibitor 10 μl/ml, phosphatase inhibitors 2 and 3 (Sigma), pH  7.2) buffer per T75 flask. The samples were centrifuged at 13 000 rpm for 15 min at 4°C. The supernatant was transferred to a new microcentrifuge tube (this is the cytoplasmic fraction). The remaining nuclear pellet in the microcentrifuge was gently washed three times with 200 ul of TLB buffer. The nuclear pellets were resuspended in TLB and the nuclear lysate samples were sonicated with a Microson XL-2000 sonicator (Misonix) 4× (6 s sonication/10s rest on ice). Samples were centrifuged at 21 130g for 15 min at 4°C. The resulting supernatant (the nuclear fraction) was then transferred to a new microcentrifuge tube. The protein concentration for each sample was determined using 595 nm wavelength OD values against a bovine serum albumin (BSA) standard.

### Immunofluorescence staining and confocal microscopy

For each transfection, 50 000 cells were seeded onto each glass slide. The cells were transfected with 1ug of each indicated plasmid and fixed and stained 24 h post transfection. Primary antibodies were incubated for overnight at 4°C (cell signaling anti-FLAG 9A3, cell signaling anti-T7 D9E1X and secondary antibodies (Alexa Fluor 488 and Alexa Fluor 488) were incubated for 1 h at room temperature. Slides were mounted with DAPI (4′,6′-diamidino-2-phenylindole), and confocal analysis was performed using Nikon Ti2 confocal microscope.

### L1 element amplification protocol (LEAP) adapted from (85,86)

Briefly, 4 million HeLa cells were seeded 16–18 h prior to transfection in T75 flasks and co-transfected with 5 ug of each indicated plasmid. 12 ul of Plus reagent (Life Technologies) in 180 ul serum free media was mixed with 24 ul Lipofectamine reagent and 76 ul serum free media (Life Technologies). The transfection cocktail was added to the cells in 6 ml of serum free media for 3 h, after which it was replaced with 10 ml of Minimum Essential Media (MEM). Approximately 48 h post-transfection, cells were washed as described above and harvested in 5 ml PBS. Cells were pelleted at 3000g for 5 min at 4°C and then lysed in 500 l of LEAP lysis buffer (1.5 mM KCl, 2.5 mM MgCl2, 5 mM Tris–Cl, 1% DOC, 1% Triton X-100, EDTA and RNAsin). After incubation on ice for 5 min, samples were centrifuged for 5 min at 3000g at 4°C to remove debris. Supernatant was layered on top of an 8.5% sucrose cushion that was layered on top of a 17% sucrose cushion, and then centrifuged at 36 500 rpm for 2 h at 4°C. Supernatant was removed, and pellet was resuspended in 100 ul deionized water supplemented with HALT protease inhibitor and RNAsin. Protein concentration was assessed using Bradford assay and samples were brought to equal protein concentrations. Samples were stored at –80°C in final 50% volume/volume glycerol. ORF2p-mediated LEAP reaction was performed using 1ug of each sample. The following primers were used to assess ORF2p-generated cDNA:

LEAP forward primer 1: AAGGACACCTGCACCCGGATGTTCATC

LEAP forward primer 2: TCCTGCGGGACCTGGAGCTGGAGAT

LEAP forward primer 3: CATCATCAAGAAGAGCGGCAACAAC

LEAP forward primer 4: CTTCAGCAAGGAGGACATCTACGC

LEAP forward primer 5: AAGATTTTCGCCACCTACAGCAGCGACAAG

LEAP reverse primer: AGTGGCACCTTCCAGGGTCAAGGAA

### Regular RT-PCR

Reverse Transcriptase System (Promega: A3500) was used according to manufacturer's instructions. Reactions were set up at 9 ul sample. Each sample was done in duplicates with one for RT+ one for RT–. The RT+ reaction was added with 1.5 ul of AMV, the RT- reaction was added with distilled water. The final volume was brought up to 20 ul with distilled water. The following samples were incubated at 70°C for 10 min. For each sample, the same set of primers in LEAP assay were used for conventional RT-PCR.

### Ruler PCR assay to measure L1Neo insertion length

G418 resistant clones were selected as described for retrotransposition assay. Individual Neo-resistant colonies were picked and expanded in 12 well plates in 500 μg/ml G418. Genomic DNA was extracted from cells generated from individual colonies. Thirty-two clones were randomly selected from each treatment and subjected to genomic DNA extraction. GoTaq-Hot start polymerase (Promega PRM5122) was used for the PCR reactions. Each PCR reaction contained 100–200 ng of genomic DNA, 2 ul of each 10uM primer, and nuclease-free water was used to bring up the total volume of 50 μl. Typical amplification conditions were: 95°C, 3 min; (95°C, 1min; 62°C, 30 s; 72°C, 1 min per kilobase) × 35 cycles; 72°C, 5 min; 4°C hold on thermocycler. Oligonucleotides used for PCR included the following: reverse primer: 5’-ATTGAACAAGATGGATTGCACGC-3’; forward primer: 5’-CAGGGATGCCCTCTCTCACCG-3’. PCR products were analyzed on 1% agarose gels.

### MELT analysis

MELT ver.2.0 package (87) was used to analyze the BWA aligned WGS data sets of endometrial, ovarian, and prostate cancer patients. MELT parsed WGS data for DRPs (defined as mates that are either aligned to different chromosomes or separated by at least 1 Mbp). DRPs were then aligned to mobile element L1.3 reference sequences using Bowtie 2, where one mate mapped to the human reference sequence and the other mapped to a L1.3 reference sequence. MELT used SRs to further refine L1 insertion-associated features and precise breakpoints. All reported sites were then merged into a VCF format file. For each individual patient, both tumor and blood/adjacent tissue WGS were assessed and compared to define the tumor specific L1 *de novo* insertions. In brief, the detected L1 insertions in tumor and blood/normal tissues were defined as true positive calls with the filter label ‘pass’. All the true positive L1 insertions found in tumor samples were cross-referenced with the ones detected in blood/normal tissues. Only the ones not detected in blood/normal samples but unique to tumor samples were considered as tumor specific L1 insertions. Tumor specific full-length L1 insertions were cross referenced with a list of reported polymorph L1s (88,89) and only the ones that have not been reported as polymorphic L1 were considered as authentic tumor specific L1 insertions.

### RNA-seq sample preparation and analysis

Cytoplasmic RNA was extracted and prepared as previously described (90). Specifically, HeLa cells were transfected with 0.8ug of indicated plasmid (PCDNA3.1+ used as control, TRIM28 WT or TRIM28 3M) and harvested at 48h post-transfection. 500 ul of lysis buffer (150 mM NaCl, 50 mM HEPES pH 7.4, 25 ug/ml digitonin with 1000 U/ml SUPERase-In RNase inhibitor) was used to lyse the cytoplasmic membrane. The mixture was incubated on ice for 5 min and then centrifuged for 2 min at 1000g at 4°C. Supernatant which contains the cytoplasmic fraction, was mixed with pre-chilled 7.5 ml of Trizol and 1.5 ml of chloroform and then centrifuged for 35 min at 4000 rpm at 4°C. The aqueous portion was transferred to 4.5 ml of chilled chloroform, mixed and centrifuged for 10 min at 4000 rpm at 4°C. The resulting aqueous portion was precipitated with 5 ml of isopropanol overnight at 80°C, centrifuged for 45 min at 4°C at 4000 rpm, washed with 10 ml of ethanol and re-suspended in RNase-free water.

Strand-specific, paired-end BGI RNA-Seq (2 × 150 bp) on polyA-selected cytoplasmic RNA was performed by BGI as previously described (90–93). RNA-Seq reads were aligned using previously reported alignment parameters (93) to the HG38 genome and the expressed L1 loci were cross-referenced with previously manually validated L1 loci determined as expressed in HeLa cells (93). These loci were then manually validated for expression in the RNA-Seq data sets generated from control, TRIM28 WT and 3M samples. RNA-sequencing fastq files were processed using Kallisto v0.46.0 (74). Sleuth v0.30.0 (Wald test) was used to calculate differential expression values including fold change (FC) and *P* value (75). These data were then used for gene set enrichment analysis (GSEA).

For gene expression normalization, GraphPad Prism 9.4 was used for analysis. All genes across 12 different samples (Control, TRIM28 WT and TRIM28 3M, 4 replicates for each treatment) were normalized between 0 and 100%. Zero was defined as the smallest value in each data set, and one hundred was defined as the largest value in each data set. All values in between were then normalized accordingly (Supplementary table S2).

## RESULTS

### Human and mouse TRIM28 both significantly increase L1 retrotransposition

**Figure 1.**
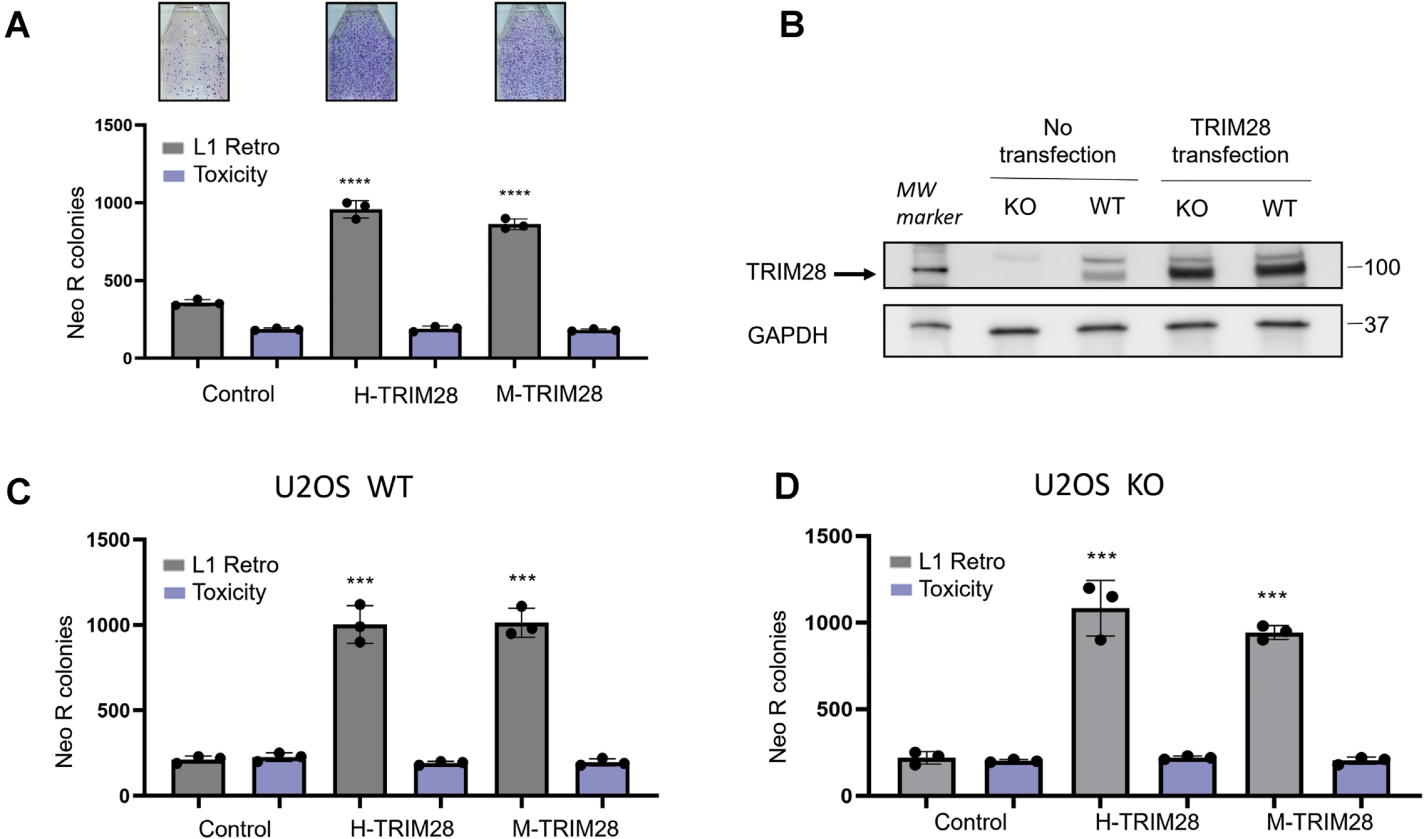
Both human TRIM28 (H-TRIM28) and mouse TRIM28 (M-TRIM28) increase L1-Neo retrotransposition. (**A**) Grey bars represent results of L1 retrotransposition assay in HeLa cells co-transfected with a plasmid expressing a Neo-tagged, full-length human wild type L1 and either an empty plasmid (control) or a plasmid expressing either H-TRIM28 or M-TRIM28. Images of flasks containing Neo-resistant (Neo R) colonies corresponding to L1Neo retrotransposition are shown above the graph. Purple bars are results of toxicity assay for which the pIRES2-EGFP vector carrying a constitutive Neo-resistant expression cassette (Neo R) is co-transfected with the control, H-TRIM28, or M-TRIM28 expressing plasmids. (**B**) Western blot analysis of endogenous TRIM28 expression in wild type U2OS cells (U2OS WT) and U2OS TRIM28 knock-out cells (U2OS KO). U2OS KO and U2OS WT cells transfected with a plasmid expressing FLAG tagged human TRIM28 are used as positive control. The lower molecular weight band (indicated by the arrow) corresponds to TRIM28. GAPDH is used as loading control. (**C**) L1 retrotransposition and toxicity assays performed in U2OS WT cells using plasmids and conditions described in (A). (**D**) L1 retrotransposition and toxicity assays performed in U2OS KO cells using plasmids and conditions described in (A). For all experiments asterisks (*) denote statistical significance between indicated experimental data points and the control (*n* = 3, *t*-test, ***P < 0.001, **** *P* < 0.0001). Dots represent number of Neo R colonies observed in individual experiments. Error bars represent the standard deviation (SD).

**Figure 2.**
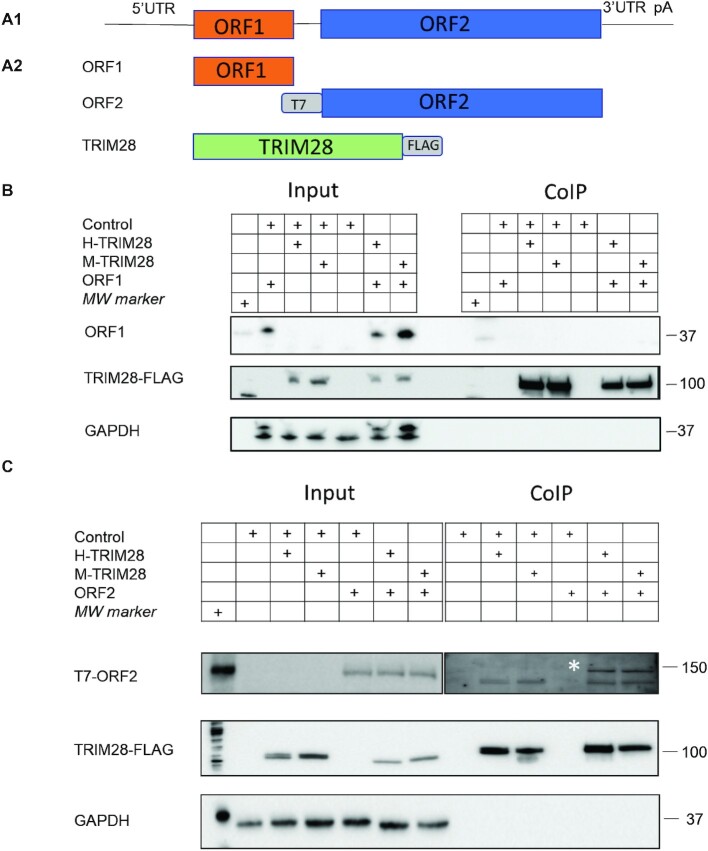
Human and Mouse TRIM28 specifically interact with human L1 ORF2 protein. (**A1**) Schematic of a full length L1, containing 5’UTR, two open reading frames (ORF1 and ORF2) and 3’UTR ending with a polyA site and a polyA tail. (**A2**) Schematic of plasmids used for co-Immunoprecipitation assay in HeLa cells. ORF1p is not tagged. T7 indicates the position of the T7 tag in the ORF2p expressing plasmid. FLAG indicates the position of the FLAG tag in the TRIM28 expressing plasmid. (**B**) Results of co-IP using lysates of HeLa cells transfected with indicated plasmids (+) and anti-FLAG-beads assessed by western blot analysis. ORF1 (the upper bands slightly above GAPDH) is detected using anti-ORF1 antibodies. TRIM28 is detected using anti-FLAG antibodies. GAPDH is used as loading control (the lower bands). Input corresponds to assessment of protein expression in whole cell lysates. CoIP corresponds to the assessment of co-IP results. (**C**) Results of co-IP using lysates of HeLa cells transfected with indicated plasmids (+) and anti-FLAG-beads assessed by western blot analysis. ORF2p is detected using anti-T7 antibodies. TRIM28 is detected using anti-FLAG antibodies. GAPDH is used as loading control. The arrow indicates a non-specific band in the input lysates that masks detection of transfected ORF2p in HeLa cells. The asterisk indicates an ORF2p-specific band.

Previous studies have oppositely shown that TRIM28 acts as either a potentially positive or negative regulator of L1 retrotransposition in HeLa-M2 cells and K562 cells (32,58). Since the amino acid sequences of the human and mouse TRIM28 proteins are highly conserved, we hypothesize that both human and mouse TRIM28 have similar effects on L1 retrotransposition. To test this hypothesis, HeLa cells were transiently transfected with a plasmid containing sequences to express human neomycin-tagged L1 (L1Neo) (81) and a plasmid containing sequences to express either full-length human or mouse TRIM28 proteins (H-TRIM28 or M-TRIM28). An empty plasmid co-transfected with the L1Neo plasmid was used as a control. Under these experimental conditions co-expression of L1Neo plasmid with either human or mouse TRIM28 expression plasmids increased L1 retrotransposition on average by 3-fold (Figure 1A, grey bars). To account for any potential effect on cell viability caused by TRIM28 overexpression, a neomycin expressing plasmid pIRES, was transiently co-transfected into HeLa cells with a control plasmid or a plasmid expressing either human or mouse TRIM28. This approach determined that there is no difference in the number of Neo-resistant colonies resulting from pIRES plasmid between the control and experimental conditions (Figure 1A, purple bars). Controls for L1 protein expression were also performed and are shown in multiple figures (Figure 2, Supplementary Figure S8). In summary, co-transfection with the wild type TRIM28 does not change L1 protein expression levels regardless of the plasmid driving their expression. Thus, expression of either H-TRIM28 or M-TRIM28 proteins significantly increased L1 retrotransposition in HeLa cells without affecting L1 protein expression levels or cell viability. Co-transfection of the TRIM28 expression plasmid with the L1Blast expression plasmid also resulted in the increase in L1 retrotransposition in HeLa cells demonstrating that this effect is not specific to the L1Neo reporter construct (Supplementary Figure S9).

To determine whether this effect can be observed in other cell lines and to determine whether TRIM28 is required for L1 retrotransposition, we generated TRIM28 knock out U2OS cells (U2OS KO) (see materials and methods) that do not express endogenous TRIM28 but support equal expression levels of transiently transfected human TRIM28 protein (Figure 1B). L1 retrotransposition assays (81) and toxicity assays (18,29) performed in U2OS wildtype (U2OS WT) and U2OS TRIM28 knockout cells (U2OS KO) under the same experimental conditions demonstrated that both H-TRIM28 and M-TRIM28 significantly increase L1 retrotransposition in these cell lines. However, the baseline level of L1 retrotransposition, reflected by Neo R colonies in the control, did not vary between the WT and KO U2OS cell lines (Figure 1C, D). These results demonstrate that endogenous TRIM28 is not required for L1 retrotransposition or that its involvement in L1 mobilization is redundant. These results also show that TRIM28 increases L1 retrotransposition in multiple human cell lines when overexpressed (Figure 1).

### Human and mouse TRIM28 interact specifically with ORF2p but not ORF1p

One possible mechanism through which TRIM28 may promote L1 retrotransposition is through interaction with L1 proteins. To test whether human and mouse TRIM28 proteins interact with human L1 ORF1 or ORF2 protein (ORF1p and ORF2p, respectively), we transiently co-expressed FLAG-tagged H-TRIM28 or M-TRIM28 with L1 ORF1p or T7-tagged L1 ORF2p and performed co-immunoprecipitation in HeLa cells using beads conjugated with anti-FLAG antibodies (Figure 2). Western blot analysis of CoIP lysates confirmed that both human and mouse TRIM28 interact with human ORF2p (Figure 2C) but not human ORF1p (Figure 2B). GAPDH is used as a loading control. These findings suggest that TRIM28 may directly or indirectly affect ORF2p function.

### Multiple domains of ORF2p interact with TRIM28

To identify the domains of the human ORF2p responsible for the interaction with TRIM28, we generated a series of T7 tagged ORF2p fragments (see materials and methods). Plasmids containing each of these fragments were individually transiently co-transfected with a plasmid expressing either a full-length H-TRIM28-FLAG or a full-length M-TRIM28-FLAG proteins. Cellular lysates collected from HeLa cells co-transfected with these combinations of plasmids were used to perform co-immunoprecipitation followed by western blot analysis (Supplementary Figure S1). This approach determined that the ZRT, C-terminus, and Cryptic domains all interact with both mouse and human TRIM28 (Supplementary Figure S1A, C, D). In contrast, the endonuclease (EN) domain of ORF2 does not bind to either human or mouse TRIM28 (Supplementary Figure S1B). However, EN expression dramatically reduces TRIM28 expression levels, which may interfere with the detection of its interactions. We also determined that the Z and RT domains can individually interact with the full-length H- and M-TRIM28 proteins in HeLa cells (Supplementary Figure S1A3). These findings identify the redundancy in interaction between TRIM28 and known ORF2p domains with yet unknown biological relevance.

### N-terminal fragments containing B box of TRIM28 increase L1-retrotransposition in HeLa cells

**Figure 3.**
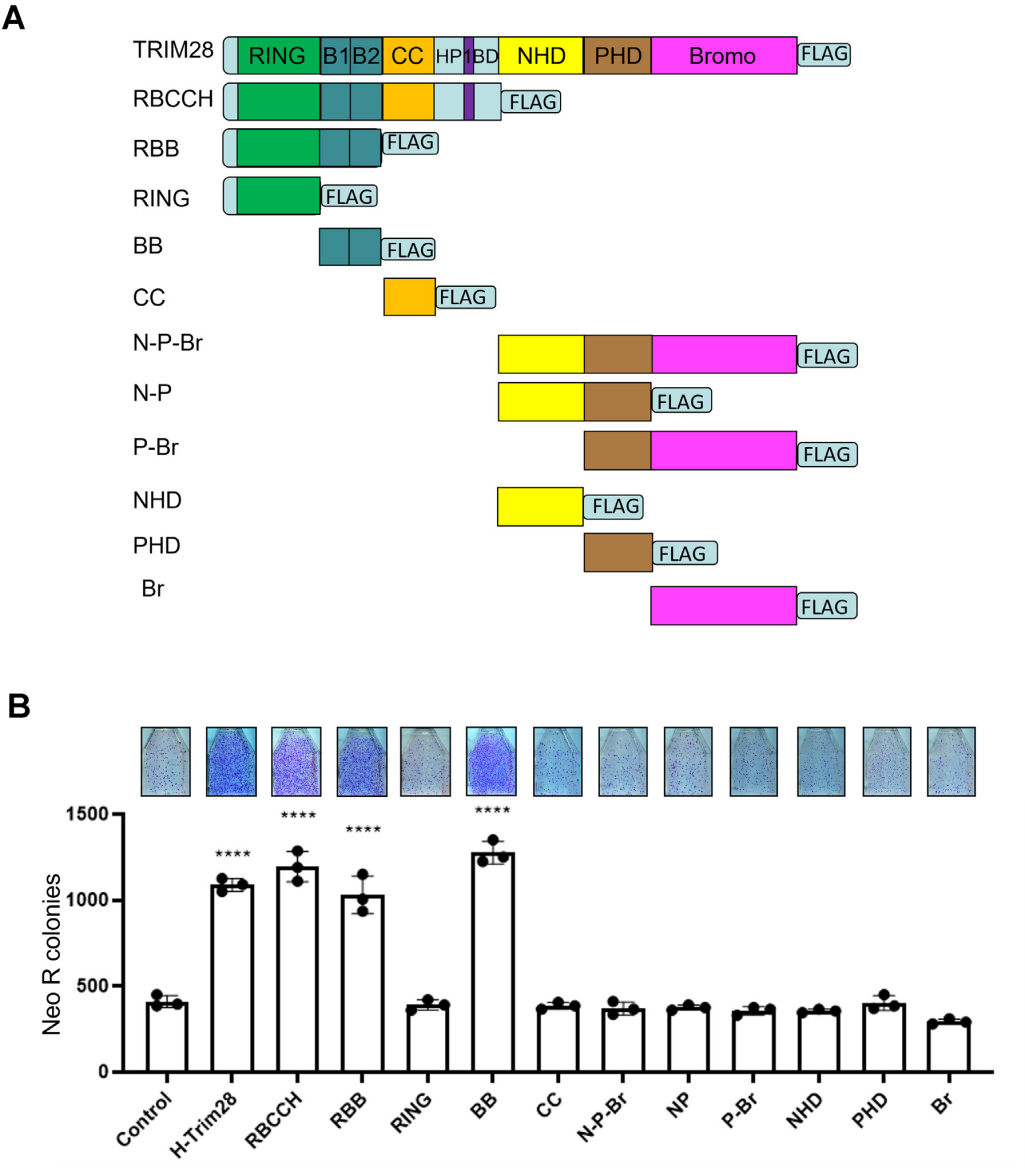
N-terminal B box containing TRIM28 fragments increase L1 retrotransposition. (**A**) Schematic of TRIM28 fragments tested in the L1 retrotransposition assay. All fragments are generated from Human TRIM28, and FLAG-tagged at the C terminus. Names of constructs are reported on the left. The amino acid coordinates corresponding to each fragment are described in materials and method. (**B**) Results of L1 retrotransposition assay in HeLa cells using plasmids depicted in A. The number of Neo R colonies resulting from co-transfection of an empty plasmid with a plasmid expressing Neo-tagged, full-length human wild type L1 is used as control (control). Images of flasks containing Neo R colonies corresponding to L1Neo retrotransposition are shown above the graph. Asterisks (*) denote statistical significance between listed constructs and the control (*n* = 3, *t*-test, **** *P* < 0.0001). Dots represent number of Neo R colonies observed in individual experiments. Error bars represent the standard deviation (SD).

To identify whether a specific part(s) of the human TRIM28 may be responsible for increasing retrotransposition of the Neo-tagged L1, we generated plasmids containing different domains of human TRIM28 protein as previously described (56,68) (Figure 3A). Plasmids containing these TRIM28 fragments were individually co-expressed with the L1Neo-expression plasmid to test their effect on L1-retrotransposition in HeLa cells. This approach determined that all N-terminal domains containing the B box domain of the human TRIM28 protein increase L1 retrotransposition as much as the full-length TRIM28 does (Figure 3B). In fact, co-transfection of the B box (BB) domain alone is sufficient to recapitulate the effect of the full-length TRIM28 in increasing L1Neo retrotransposition (Figure 3B). In contrast, fragments that do not contain the BB domain were unable to stimulate L1 mobilization, indicating that the BB domain is the critical part of TRIM28 for the observed enhancement of L1 retrotransposition (Figure 3B).

To determine whether the effect of TRIM28 fragments on L1Neo retrotransposition is dependent or independent of their ability to interact with L1 ORF2p, we performed a series of Co-IP experiments in HeLa cells using TRIM28 fragments and the ZRT fragment of human ORF2p. The ZRT construct is chosen to screen these interactions because the ZRT protein fragment contains an important enzymatic activity of the ORF2p and is easily detectable compared to other ORF2p fragments and the full-length L1 ORF2p. Western blot analysis of co-IP lysates demonstrates that all TRIM28 N- and C-terminal domains can interact with ZRT to a certain extent (Supplementary Figure S2A, B). In addition, we assessed cellular localization of ORF2p and TRIM28 using immunofluorescence and confocal microscopy (Supplementary Figure S3) of cells transfected with plasmids expressing ORF2p (FITC green) and full-length or truncated TRIM28 (TRITC red). The results demonstrated the presence of cells with yellow signal corresponding to the superimposed signal of ORF2p and TRIM28 proteins supporting colocalization of the respective proteins. (Supplementary Figure S3A–G, merged panel). Although RING, CC and all C-terminal domains interact with ZRT, none of them increases L1-retrotransposition as does full-length TRIM28, B box, or B box-containing fragments (Figure 3B). These results demonstrate that the interaction between the ZRT or ORF2p and TRIM28 fragments by itself is not sufficient to increase L1Neo retrotransposition.

To further characterize the TRIM28-ZRT interaction we evaluated subcellular localization of this interaction using western blot analysis. HeLa cells transfected with the TRIM28 and ZRT expression plasmids separately or together were fractionated to separate nuclear and cytoplasmic parts as previously described (83,84). Each of these fractions was then used for co-IP and western blot analysis. Consistent with the results in the Supplemental Figure S3, this approach detected TRIM28 and ZRT proteins and their interaction in both cellular compartments, with the more robust steady state interaction of ZRT with TRIM28 being detected in the nuclear fraction (Supplementary Figure S4). To test the involvement of RNA in this interaction we performed co-immunoprecipitation analysis with RNase A treatment (Supplementary Figure S5). We confirmed RNA degradation by running extracted RNA samples from cells co-expressing TRIM28 and ORF2p on an RNA gel (Supplementary Figure S5A). Our result shows that the interaction between ORF2p and TRIM28 is maintained in the nuclease treated samples (Supplementary Figure S5B). These data support an RNA-independent ORF2p–TRIM28 interaction, with the caveat that the piece of RNA mediating the protein interaction could be inaccessible during RNase treatment as it could be potentially protected by the protein complex.

### The 3 amino acids reported to interfere with TRIM28 multimerization are critical for increasing L1 retrotransposition

**Figure 4.**
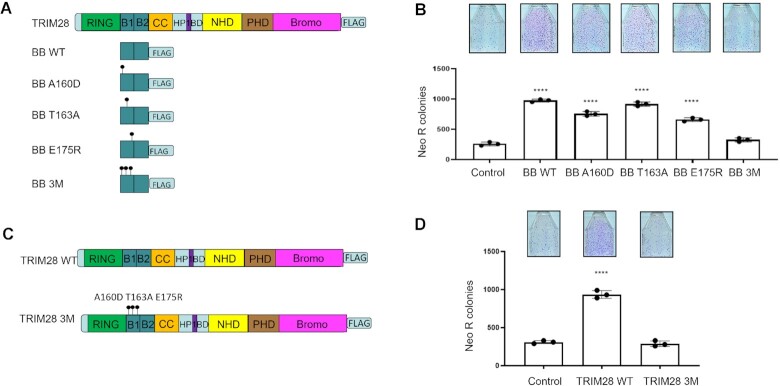
Amino acids involved in TRIM28 multimerization are required for its ability to increase L1 retrotransposition. (**A**) Schematic of TRIM28 B box variants. BB is B box, WT is wild type, single or triple mutations are indicated using single letter amino acid code and amino acid position in the human wt TRIM28 protein. (**B**) L1 retrotransposition result. HeLa cells are transiently co-transfected with plasmids expressing human neomycin tagged L1 (L1Neo) and indicated TRIM28 BB variants. Images of flasks containing Neo R colonies corresponding to L1Neo retrotransposition are shown above the graph. (**C**) Schematic of full-length wild type TRIM28 (TRIM28 WT) and triple mutant TRIM28 (TRIM28 3M). Amino acid mutations are noted as described in A. (**D**) L1 retrotransposition result. HeLa cells are transiently co-transfected with plasmids expressing human neomycin tagged L1 (L1Neo) and indicated full-length TRIM28 variants. Images of flasks containing Neo R colonies corresponding to L1Neo retrotransposition are shown above the graph. Asterisks (*) denote statistical significance between listed constructs and the control (*n* = 3, t-test, ****P < 0.0001). Dots represent number of Neo R colonies observed in individual experiments. Error bars represent the standard deviation (SD).

It is reported that the higher-order assembly of TRIM28 dimers is dependent on B box interactions, which could be abolished by three characterized mutations A160D/T163A/E175R (56). To investigate the effect of these mutations on the B box ability to increase L1 retrotransposition, we generated plasmids expressing TRIM28 B box domain containing single (BB A160D, BB T163A, or BB E175R) or triple mutations (BB 3M) (Figure 4A). Only the triple mutant variant, BB 3M, has lost the ability to enhance L1 retrotransposition in HeLa cells, whereas all the single mutant B box variants still increase L1 retrotransposition relative to the control (Figure 4B). Western blot analysis demonstrates that the BB 3M is expressed at the same steady state levels as the wild type and single mutant B box proteins (Supplementary Figure S6 A2 input side). Co-IP followed by western blot analysis shows that all mutant B box proteins interact with the ZRT protein (Supplementary Figure S6 A2 co-IP side). These results demonstrate that the inability of BB 3M to increase L1-retrotransposition is independent of its interaction with ZRT.

Consistent with our findings with the triple mutant B box, full-length triple mutant TRIM28 (TRIM28 3M) is unable to increase L1 retrotransposition compared to TRIM28 WT (Figure 4C, D). Western blot analysis showed that there is no difference in the ORF1p expression when TRIM28, BB, TRIM28 3M, or BB 3M are co-transfected with the L1Neo expression plasmid (Supplementary Figure S8). Consistent effect of TRIM28 WT and BB WT on L1 retrotransposition results were obtained with L1-blasticidin expression plasmid (Supplementary Figure S9). Western blot analysis determined that the TRIM28 3M is expressed at the same steady state levels as TRIM28 WT (Supplementary Figure S6B2 input side). Co-IP followed by western blot analysis showed that TRIM28 3M retains interaction with the ORF2, ZRT, and C-term proteins (Supplementary Figure S6B–D). These results demonstrate that the loss of ability of TRIM28 3M to increase L1 retrotransposition is not caused by the loss of its interaction with the L1 ORF2 protein or any of the ORF2p fragments, although we cannot exclude the possibility that TRIM28 WT and 3M may interact with different parts of ORF2, ZRT or C-term proteins. As expected, TRIM28 and its B box domain increase L1 retrotransposition in both U2OS WT and TRIM28 KO cell lines, whereas neither BB 3M nor TRIM28 3M can enhance L1 retrotransposition in these cell lines (Supplementary Figure S7A, B).

### Multiple cellular pathways involved in DNA damage response are affected by TRIM28 overexpression in HeLa cells

**Figure 5.**
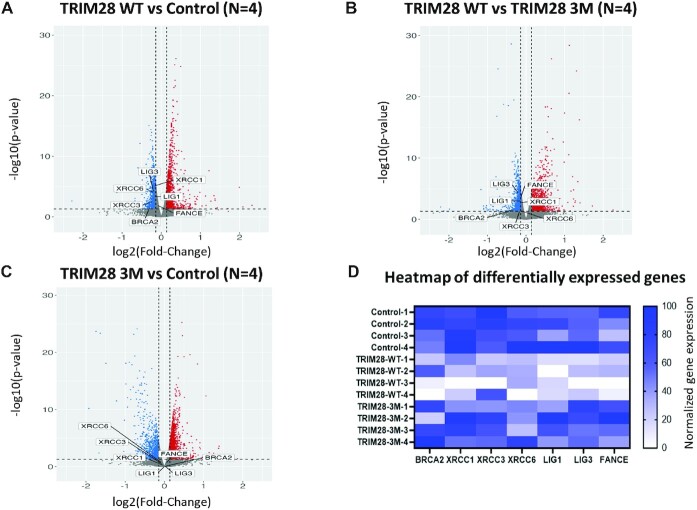
Results of analysis of differentially expressed genes in HeLa cells over-expressing control plasmid, TRIM28 WT, or TRIM28 3M. (**A**) The volcano plot shows differentially expressed genes in HeLa cells overexpressing TRIM28 WT versus Control. The horizontal grey line indicates *P* = 0.05. A fold change cutoff of 1.1 in the graph is shown as the dashed lines running parallel to the y-axis. Multiple DNA repair genes are significantly downregulated in TRIM28 WT, compared to Control. (**B**) The volcano plot shows differentially expressed genes in HeLa cells overexpressing TRIM28 WT versus TRIM28 3M. The horizontal grey line indicates *P* = 0.05. A fold change cutoff of 1.1 in the graph is shown as the dashed lines running parallel to the y-axis. Multiple DNA repair genes are significantly downregulated in TRIM28 WT, compared to TRIM28 3M. (**C**) The volcano plot shows differentially expressed genes in HeLa cells overexpressing TRIM28 3M versus Control. The horizontal grey line indicates *P* = 0.05 in Wald test. A fold change cutoff of 1.1 in the graph is shown as the dashed lines running parallel to the y-axis. The indicated DNA repair genes are not differentially expressed in TRIM28 3M compared to Control. (**D**) Heatmap of normalized expression of individual DNA repair genes that are significantly differentially expressed in HeLa cells transfected with TRIM28 WT compared to the control and TRIM28 3M expression plasmids. (Wald test, *P*< 0.05).

Having determined that the effect of TRIM28 on L1 retrotransposition is not dependent on its interaction with ORF2p, we proceeded to determine whether TRIM28 overexpression alters cellular pathways that may be involved in modulating L1 retrotransposition. We performed paired-end, stranded RNA-Seq analysis of cytoplasmic RNA extracted from HeLa cells 48 h post transfection with control, TRIM28 WT, or TRIM28 3M expression plasmids (*N* = 4 for each condition). This time point was chosen based on previous findings that L1 retrotransposition in this assay is detectable after 36 h post-transfection (94). We performed transcriptomic profiling of our RNA-sequencing data using Kallisto and Sleuth to measure and quantify differences in gene expression among the 3 indicated comparisons: TRIM28 WT versus Control; TRIM28 WT vs TRIM28 3M and TRIM28 3M versus Control. Sleuth v0.30.0 (Wald test) was used to calculate differential expression values including fold change (FC) and the p value. Multiple DNA repair genes are found to be significantly downregulated upon TRIM28 WT overexpression compared to Control and TRIM28 3M samples (Figure 5A-D, Supplementary table S1 and S2), including genes suppressing L1 retrotransposition (28–32). A heat map reflecting a decrease in the relative expression of BRCA2, XRCC1, XRCC3, LIG1, LIG3 and FANCE genes in the TRIM28 WT samples compared to the Control and TRIM28 3M samples is shown (Figure 5D) (Wald test, *P* < 0.05). We used RNA-Seq by Expectation-Maximization (RSEM) analysis and gene set enrichment analysis (GSEA) to detect cellular pathways altered in the TRIM28 WT samples compared to the Control and TRIM28 3M samples (*N* = 4 each). This approach also identified DNA repair as a significantly downregulated pathway when TRIM28 WT was overexpressed (Supplementary Figure S10). Functional NER pathway has been previously established to suppress L1 retrotransposition, while defects in NER genes resulted in the increase in L1 retrotransposition (30). This finding supports that TRIM28-induced changes in cellular DNA repair may be contributing to the TRIM28-associated increase in L1 mobilization observed in this study.

TRIM28 has been previously reported to suppress expression of endogenous retroviruses and LINE-1 (51,55,57,95,96). We used our RNA-Seq data sets to determine whether there are any changes in the number or levels of expression of endogenous L1 loci in HeLa cells overexpressing TRIM28 WT or 3M proteins. Cytoplasmic RNA-Seq reads were aligned to the HG38 genome using stringent Bowtie alignment parameters and cross-referenced with previously manual-curated full-length L1 loci expressed in HeLa cells as previously reported (91,93). Our results demonstrate that 48h post-transfection there is no significant difference in the number or levels of expressed L1 loci in the control HeLa cells transfected with empty plasmid and HeLa cells transiently overexpressing TRIM28 WT or TRIM28 3M proteins (Supplementary Figure S11A and B). These results show that under these experimental conditions TRIM28 overexpression does not significantly affect patterns or levels of endogenous L1 mRNA expression.

### B boxes of the other two members in class VI TRIM protein families, TRIM24 and TRIM33, also increase L1 retrotransposition

**Figure 6.**
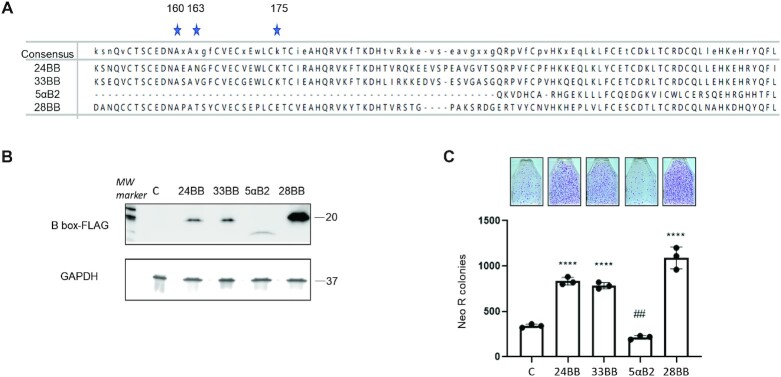
Effect of TRIM24, TRIM33 and TRIM5α B boxes on L1 retrotransposition in HeLa cells. (**A**) Amino acid sequence alignment of TRIM24, TRIM33, TRIM5α and TRIM28 B boxes (24BB, 33BB, 5αB2 and 28BB, respectively) is performed using Clustal Omega method. Stars indicate the positions of the three amino acids where TRIM28 B box mutations are introduced (shown in Figure 4C). (**B**) Western blot analysis of the proteins expressed by B box constructs described in A. All indicated B box constructs are FLAG tagged on the C terminus to allow their detection. GAPDH is used as loading control. (**C**) Results of L1 retrotransposition assay in HeLa cells co-transfected with L1Neo-expression plasmid and one of the plasmids containing constructs shown in A. Images of flasks containing Neo R colonies corresponding to L1Neo retrotransposition are shown above the graph. Asterisks (*) denote statistical significance between listed constructs and the control (*n* = 3, *t*-test, **** *P* < 0.0001, ## *P* = 0.0016) Dots represent number of Neo R colonies observed in individual experiments. Error bars represent the standard deviation (SD).

The amino acid sequences of the Class VI TRIM protein B Boxes are highly conserved (69), (Figure 6A). Therefore, we hypothesized that the B boxes from the other two members in this family, TRIM24 and TRIM33, can also increase L1 retrotransposition. The B box domains of TRIM28, TRIM33, and TRIM24 are composed of B1 and B2 sequence. By contrast, the B box of TRIM5α, a member of the Class IV TRIM family that only has B2 sequence (60,97), has been reported to contribute critically to the TRIM5α -associated restriction of L1 retrotransposition (49). To test this hypothesis, plasmids expressing B boxes from either TRIM24 (24BB), TRIM28 (28BB), TRIM33 (33BB), or TRIM5α B2 box (5αB2) were used in the L1Neo retrotransposition assay. Both 24BB and 33BB significantly increased L1 retrotransposition (Figure 6C) despite lower expression levels than the expression levels of the TRIM28 B box (28BB) (Figure 6B). Consistent with the reported observation (49), TRIM5α B2 box (5α B2) decreased L1 retrotransposition (*P* = 0.0016) (Figure 6C) (Figure 6B). Based on these findings, we sought to determine individual contributions of B1 and B2 sequences to the TRIM28-induced increase in L1 retrotransposition. Unfortunately, plasmids containing these sequences yield undetectable levels of B1 and B2 protein expression, which prevented us from testing their effect on L1 mobilization. Similarly, expression of the full length TRIM24 and TRIM33 proteins were extremely low or undetectable, consistent with previous reports of short half-life of these proteins (98). These results show that B boxes of the Class VI TRIM proteins increase L1 retrotransposition, supporting a possibility of redundancy in their effect on L1 mobilization.

### Overexpression of TRIM28 or its BB domain leads to shorter cDNA products generated by the L1 ORF2p

Our results show that TRIM28 protein interacts with L1 ORF2p but not ORF1p (Figure 2). This observation combined with the TRIM28 protein interaction with the ZRT fragment (Supplementary Figure S1A3) support that TRIM28 may affect ORF2p enzymatic functions. We sought to determine whether the interaction of TRIM28 with ORF2p affects the ability of ORF2p to reverse transcribe its mRNA. We hypothesized that longer cDNA products could be producing more Neo- or Blast resistant colonies in our retrotransposition assay because their full-length reporter cassettes are required for colony formation.

**Figure 7.**
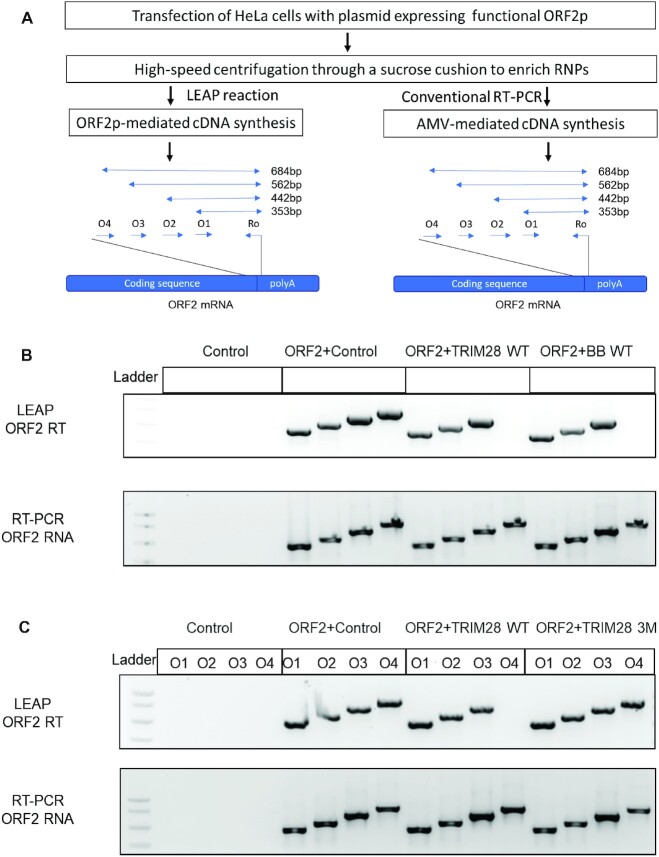
Analysis of cDNA products generated by the ORF2p in HeLa cells transfected with wild-type or mutant TRIM28 or TRIM28 B Box. (**A**) Flow chart of the LEAP assay adapted from (85,86). ORF2p-generated cDNA is detected by PCR with a step wise set of ORF2 sequence specific forward primers (O1-O4) and a reverse primer Ro. In parallel, conventional RT-PCR was performed with the same set of step wise ORF2 primers. O1-O4: forward ORF2 specific primers. The expected length of PCR products is shown on the right. (**B**, **C**) LEAP samples are prepared by harvesting HeLa cells 48h post-transfection with indicated constructs and analyzed with indicated sets of primers. Control is LEAP prep on cells transfected with the empty plasmid (i.e. no ORF2p expression). RNA integrity in LEAP preps is assessed with the same set of ORF2 specific primers shown in A. A PCR product expected to be produced with O4 primer is absent in cells expressing WT full-length H-TRIM28 (TRIM28 WT) or WT B box (BB WT). Mutations of three amino acids responsible for multimerization (TRIM28 3M) eliminate this effect.

The RT activity of ORF2p *in vitro* can be analyzed using the well-established L1 element amplification protocol (LEAP) (85,86,99). In short, HeLa cells co-transfected with T7 tagged ORF2p expression plasmid and a plasmid expressing either TRIM28 WT, BB WT, BB 3M, TRIM28 3M, or empty plasmid were harvested 48 h post transfection for ORF2 RNPs purification. Following ultracentrifugation of the lysates through a sucrose cushion, ORF2 mRNA was reverse transcribed by the ORF2p present in the purified RNPs in a LEAP reaction using ORF2 mRNA specific primer as described (34,78). To analyze the resulting cDNA, we used a series of ORF2 sequence-specific, stepwise primer sets designed to amplify cDNA products varying in length from 353 bp to 684 bp (Figure 7A). This approach shows that shorter cDNA transcripts are generated by the ORF2p in LEAP reactions containing RNPs produced from HeLa cells expressing TRIM28 WT or BB WT compared to the cDNA detected in LEAP reactions containing RNPs generated from HeLa cells expressing TRIM28 3M or BB 3M (Figure 7B, C; Supplementary Figure S12). To confirm that this effect is specific to the L1 ORF2p RT, the same sets of ORF2-specific primers were used in conventional RT-PCR reactions performed by Avian Myeloblastosis Virus (AMV) reverse transcriptase (Figure 7A). This result demonstrates that cDNA products of all lengths were detected in each reaction containing ORF2 RNA regardless of the HeLa lysate origin (Figure 7B, C; Supplementary Figure S10). Additionally, we tested another forward primer (O5) that is expected to produce an 804bp product in the LEAP or conventional RT reactions (Supplementary Figure S13A). As expected, the 804bp cDNA product was not detected in LEAP reactions when either TRIM28 WT or BB WT is over-expressed but was present in LEAP reactions when either TRIM28 3M or BB 3M is overexpressed (Supplementary Figure S13B). This product was detected in all ORF2 mRNA containing samples when the conventional RT-PCR analysis was performed (Supplementary Figure S13B). In parallel, the purified RNPs were subjected to western blot analysis to assess ORF2p levels. This approach determined that the expression levels of the ORF2p are the same in all samples (Supplementary Figure S14). This finding supports that TRIM28 through its B box domain has a pleotropic effect on multiple steps in the L1 amplification cycle in cultured cells.

### Overexpression of TRIM28 WT leads to shorter *de novo* L1Neo insertions

**Figure 8.**
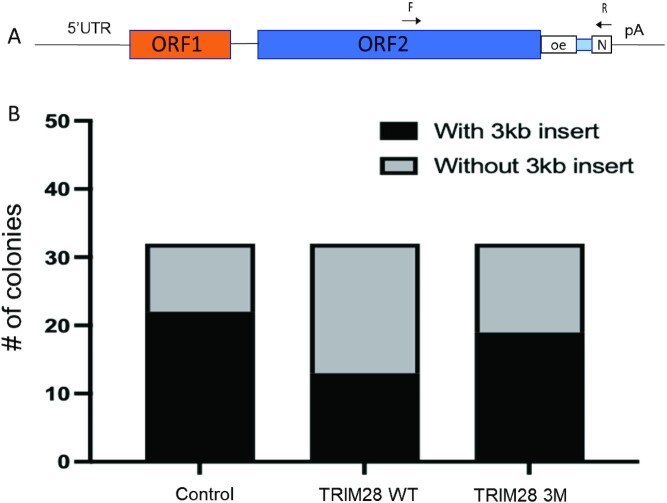
Analysis of L1 insertion in genomic DNA using ruler PCR assays (33). (**A**) Schematic of the ruler PCR assay adapted from (33). Three kilobase ruler PCR was performed using primers covering the 3kb target band. The position of primers relative to the L1 vector are shown. (**B**) PCR on genomic DNA sequence of HeLa cells transfected with L1-neo constructs was performed. Forward primer (F) and reverse primer (R) are applied. The L1-neo plasmid gives a band at 3771 bp, while the spliced L1-neo insertion gives a band at 2864 bp. Thirty-two clones were randomly picked and subjected to genomic DNA extraction. Left, number of clones with or without the spliced 3kb L1-Neo insertion in HeLa cells co-expressing L1-Neo construct and the control plasmid (PCDNA 3.1 empty vector). Middle, number of clones with or without the spliced 3kb L1-Neo insertion in HeLa cells co-expressing L1-Neo construct and TRIM28 WT plasmid. Right, number of clones with or without the spliced 3kb L1-Neo insertion in HeLa cells co-expressing L1-Neo construct and TRIM28 3M plasmid.

To confirm that our LEAP results, which are obtained using cytoplasmic RNPs, are relevant to L1 retrotransposition products, we performed a ruler PCR assay with the reported 3 kb primers (33). This size was chosen for the analysis because we used DNA extracted from the Neo resistant colonies, which must contain a full-length Neo cassette to be formed. This analysis shows that TRIM28 WT co-expression with the L1 Neo results in significantly fewer colonies containing the 3 kb PCR products (13/32), compared to the control (22/32) and the TRIM28 3M (19/32) (Figure 8, Supplementary Figure S15). Fisher's exact test shows significantly fewer 3 kb positive clones among the TRIM28 WT colonies compared to the Control colonies (*P* = 0.04). Although no significant differences were detected in TRIM28 WT versus 3M (*P* = 0.2), the 3M group was also not different from the Control group and there is a trend of fewer 3 kb positive clones being formed when TRIM28 WT is overexpressed compared to the 3M construct (13/32 versus 19/32, respectively). Consistent with our findings with the LEAP assay, TRIM28 WT overexpression leads to shorter de novo L1Neo inserts, compared to the control or TRIM28 3M overexpression.

### Shorter *de novo* L1 inserts are observed in cancer samples with high TRIM28 mRNA expression

**Figure 9.**
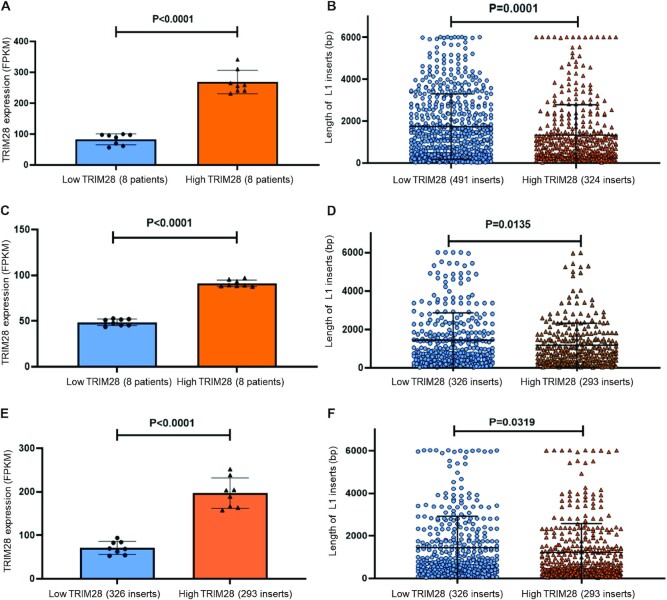
Analysis of length of tumor specific L1 insertions in WGS data set collected from endometrial, prostate, and ovarian cancer patients. (**A**) Sixteen patients with endometrial cancer are selected and grouped into two groups (*n* = 8) according to high or low TRIM28 mRNA expression levels (*t*-test, *P* < 0.0001). (**B**) Length of tumor specific *de novo* L1 inserts in endometrial cancer patients is significantly shorter in high TRIM28 group (*n* = 324) than low TRIM28 group (*n* = 491), *t*-test, *P*< 0.0001. (**C**) Sixteen patients with prostate cancer are selected and grouped into two groups (*n* = 8) according to high or low TRIM28 mRNA expression levels (*t*-test, *P* < 0.0001). (**D**) Length of tumor specific *de* novo L1 inserts in prostate cancer patients is significantly shorter in high TRIM28 group (*n* = 293) than low TRIM28 group (*n* = 326), *t*-test, *P* = 0.0135. (**E**) Sixteen patients with ovarian cancer are selected and grouped into two groups (*n* = 8) according to high or low TRIM28 mRNA expression levels (*t*-test, *P* < 0.0001). (**F**) Length of tumor specific *de novo* L1 inserts in ovarian cancer patients is significantly shorter in high TRIM28 group (*n* = 383) than low TRIM28 group (*n* = 386), *t*-test, *P* = 0.0319. For each individual figure, error bar represents the standard deviation (SD).

While a loss of function TRIM28 mutations are rarely observed in human tumors (100), its expression levels are increased in many types of human cancer (61). Higher levels of TRIM28 expression are often associated with poor diseases outcome (72–76). It is established that the B box domain mediated TRIM28 oligomerization is concentration dependent (56). To investigate whether our findings in cultured cells are relevant *in vivo*, we identified patients with endometrial cancer, prostate cancer, and ovarian cancer using GDC Legacy database. Patients within each cancer group were separated in to two groups, with 8 patients demonstrating ‘High’ and 8 patients exhibiting ‘Low’ TRIM28 mRNA expression according to the reported TRIM28 mRNA expression in their tumors (101). This stratification in endometrial cancer, prostate cancer and ovarian cancer patients revealed a 3.2-, 1.9- and 2.8-fold average difference of TRIM28 mRNA expression levels, respectively, between the ‘High’ and ‘Low’ groups of patients (Figure 9).

In addition to the TRIM28 expression levels, other inclusion criteria for the two groups within each cancer type are sex, age, race and availability of WGS bam files generated from blood/normal and cancer samples collected from the same patient. The two groups of patients with endometrial cancer have comparable age range (on average 63.7 years old for Low TRIM28 group, 70 years old for High TRIM28 group, respectively), and so do the patients with prostate cancer (on average 59 years old for High TRIM28 group and 56 years old for Low TRIM28 group, respectively) and ovarian cancer (on average 63.7 years old for Low TRIM28 group, 60.3 years old for High TRIM28 group, respectively)(Supplementary Figure S16). All patients are gender-matched due to the sex-specific organ of disease origin. The race compositions are comparable for each type of cancer (for endometrial cancer patients, each group has two African American, one pacific islander and five white patients; for prostate cancer, all patients are white; for ovarian cancer, most patients are white except one Asian and one African American in Low TRIM28 group).

MELT analysis was performed as described (87) on WGS files generated from blood/normal and cancer samples of each patient. *De novo* tumor specific L1 insertions were identified as present in the tumor WGS but not in blood/normal WGS and meeting MELT criteria of *de novo* L1 inserts as described (87). This approach identified hundreds of L1 inserts in the tumor samples of ‘High’ and ‘Low’ TRIM28 groups for each of the three cancer types (Figure 9A–F). Unpaired two-sample t test determined that there is no difference in the number of inserts per patient between the High and Low TRIM28 expression groups in any type of cancer patients (Supplementary Figure S9). Based on the mean value of the number of inserts in each group and standard deviation values of the two groups of patients we calculated that at least 142 patients per each TRIM28 expression group are needed to determine whether there is a difference in the number of *de novo* L1 inserts per patient between these two groups (power of 0.80 and alpha of 0.05). Based on the described inclusion criteria, we could not identify sufficient number of patients per group to conclusively determine whether TRIM28 expression levels affect the number of de novo L1 inserts per patient. However, the large number of total *de novo* L1 inserts identified in each group of patients has allowed us to compare their lengths. Unpaired two-sample t test analysis determined that *de novo* L1 insertions are significantly shorter in the ‘High’ TRIM28 expression group compared to the ‘Low’ TRIM28 expression group of patients in each cancer cohort (Figure 9). We also performed the Mann–Whitney *U* test, which is a non-parametric alternative to the t-test for cases when the sampling distribution of the population mean is not or may not be normally distributed. The median values are 1458 bp versus 876 bp, 886 bp versus 832 bp and 686 bp versus 807 bp for the low versus high TRIM28 group of patients with endometrial cancer, prostate cancer and ovarian cancer, respectively. Consistent with the unpaired t test analysis, Mann–Whitney *U* test comparing the median also shows significant differences between High and Low TRIM28 expression groups of endometrial and ovarian cancer patients (*P* < 0.0001 for endometrial cancer, *P* = 0.0114 for ovarian cancer). These values are calculated using all tumor-specific L1 inserts, which include those that may have happened before the increase in the TRIM28 expression occurred in any given tumor. The number of full-length L1 inserts in each group was too small to analyze them as a separate category. These patient-based findings agree with our findings in HeLa cells using LEAP assays (Figure 7B, C) as well as a ruler PCR assay (Figure 8) and demonstrates that variation in TRIM28 expression levels *in vivo* may influence the length of *de novo* L1 inserts in at least three different cancer types as well as in both sexes.

## DISCUSSION

Many cellular genes have been identified to modulate almost every known step of the L1 amplification cycle (47,102). Not surprisingly, many cellular genes are required for L1 retrotransposition as the two L1-encoded proteins only contain limited functional and enzymatic activities. Among this group are genes generating proteins involved in basic processes of gene expression such as polymerase II transcription, mRNA biogenesis, and mRNA translation (6,24,103–106). Some of the genes necessary for L1 integration, such as ATM, PARP1, PARP2 and RPA complex (107,108), function in DNA repair because L1 integration involves DNA breaks, which need to be sensed and repaired to complete *de novo* integration. The other large group of cellular genes includes those that function as suppressors of L1 mRNA expression, translation, or retrotransposition (47,102). These are genes involved in DNA methylation (DNMT1, DNMT3 etc.) and heterochromatin formation (TRIM28, HUSH, SETDB1 etc.), mRNA biogenesis (splicing and polyadenylation complexes, MOV10, APOBECs) and DNA repair (genes involved in NER and p53 pathways) (28–32,44–46,102). Identification of this group of cellular factors led to the current thinking that cells must have evolved this redundancy in suppressing L1 expression and integration to reduce damage associated with L1 endonuclease and reverse transcriptase activities. Recently, a handful of cellular genes were recognized as putative enhancers of L1 retrotransposition (32,58,106,108,109). Here, we identify TRIM28 as a cellular gene, increased expression of which enhances L1 retrotransposition and results in generation of shorter de novo inserts through its repressive effect on the ORF2p-mediated cDNA synthesis.

It has been established that TRIM28 and several other TRIM proteins epigenetically silence expression of endogenous retroviruses (50,110–112). TRIM28-mediated suppression of promoters of older L1 subfamilies is achieved through the established function of TRIM28 in the formation of heterochromatin (55,57,96). However, it is less clear whether TRIM28 binding to L1 promoters affects L1 expression because some analysis of L1 expression were performed using RT-qPCR (54,55). This methodology does not reliably distinguish between passive transcription and L1 expression (91,113). TRIM28 is upregulated in many types of cancer where endogenous L1 mRNA expression is also high (61), suggesting that its suppressive effect on L1 expression may be context specific. In this study, we detected no significant differences in the number of endogenously expressed L1 loci nor the levels of L1 mRNA expression upon TRIM28 overexpression (Supplementary Figure S8) using our previously reported bioinformatics approach (91). However, we cannot rule out the possibility that 48 h of HeLa cells exposure to exogenous TRIM28 may not be sufficient to establish suppressive chromatin state to produce measurable changes in endogenous L1 mRNA expression. Furthermore, overexpression of TRIM28 alone may not be sufficient to silence L1 mRNA expression because other cellular proteins, such as SETDB1 and NuRD, are also needed (59,68,70). Additionally, presence of un-transfected cells in our experiment creates background that may interfere with the sensitivity of detection. Together these findings support that the effect of TRIM28 on L1 mRNA expression is not coupled to its dual role in L1 retrotransposition identified in this study.

In this study we discovered that TRIM28 increases L1 retrotransposition and interferes with cDNA synthesis (Figures 1 and 7). Both effects are dependent on the B box of TRIM28. The B box domain-dependent increase in L1 retrotransposition in HeLa and U2OS cells (Figure 3, Supplementary Figure S5) is consistent with TRIM28 being identified as a putative activator of L1 retrotransposition by a screen-based assay in HeLa M2 cells (32). Our data also demonstrate that TRIM28 is not required for L1 retrotransposition because TRIM28 KO U2OS cells support comparable levels of L1 retrotransposition to the WT cells (Figure 1C, D). The fact that both mouse and human TRIM28 proteins promote L1 retrotransposition in human cancer cells suggests some degree of evolutionary conservation in TRIM28 functioning as an activator of L1 mobilization (Figure 1). We do not know the exact mechanism underlying this increase because the function of B box-dependent formation of higher degree TRIM28 oligomers is poorly understood. However, our findings that TRIM28 overexpression affects multiple cellular pathways, including those related to DNA damage response and DNA repair (Figure 5, Supplementary table S1 and S2), is consistent with the published results (114–117). Some DNA repair genes known or speculated to modulate L1 mobilization (30,118), are downregulated in cells overexpressing TRIM28 WT compared to the cells overexpressing the control plasmid and mutant TRIM28 (Figure 4D). For example, detected significant downregulation of NER pathway and BRCA2 genes could alone explain an increase in L1 retrotransposition, although follow up studies will be needed to confirm these possibilities.

TRIM28 is a multifunctional protein, but its ability to stimulate L1 retrotransposition requires only the oligomer-forming B box, while other enzymatic activities and functional properties are likely not involved (Figure 3). TRIM24 and TRIM33 are close homologs of TRIM28 and belong to the same TRIM protein subfamily Class VI (60). Their highly conserved B box domains that also increase L1 retrotransposition (Figure 6) are composed of B1 and B2 boxes. In contrast, TRIM5α B box domain, which only contains a B2 box, suppresses L1 retrotransposition (49), (Figure 6). Although we have not been able to determine whether B1 and B2 box from different TRIM proteins individually and differentially affect L1 retrotransposition because of the undetectable levels of their expression, it is possible that these boxes may differentially affect L1 retrotransposition. Together our results demonstrate functional redundancy of different Class VI TRIM proteins in increasing L1 mobilization. These results also establish that oligomerization of TRIM proteins from different TRIM classes can have opposing effects on L1 mobilization.

Our data reveal that in addition to its impact on L1 retrotransposition, overexpression of TRIM28 or its B box affects ORF2p-mediated cDNA synthesis, leading to shorter products (Figure 7B). This effect also requires functional wild type B box (Figure 7C, Supplementary Figure S6). This finding suggests that TRIM28 associated stimulation of L1 retrotransposition (Figure 1) may be underestimated in our experiments because the retrotransposition assay relies on incorporation of the entire neomycin or blasticidine resistance cassettes for detection of *de novo* L1 inserts. It is worth noting that the LEAP assay measures the ability of ORF2p to generate cDNA in the cytoplasm rather than in the nucleus, where it likely takes place during retrotransposition of both endogenous and exogenous L1s. However, our co-IP results demonstrate that TRIM28 directly or indirectly interacts with L1 ORF2p in both the nucleus and cytoplasm (Supplementary Figure S3). The validity and relevance of our LEAP assay findings in HeLa cells to L1 integration is confirmed by the ruler PCR assay. Consistent with the LEAP results, ruler PCR demonstrated that TRIM28 WT overexpression produces fewer Neo-resistant colonies containing 3kb insertions (Figure 8, Supplementary Figure S15). Consistent with the findings in cultured cells, the length of *de novo* L1 inserts in tumor samples collected from patients with high levels of TRIM28 expression is significantly shorter than in patients with low TRIM28 expression (Figure 8). This significant difference between the two groups was observed in three different cancer types in male (prostate cancer) and female (endometrial and ovarian cancers) patients. It is worth pointing out that the exact timing of TRIM28 upregulation in individual patients is not known, thus the average length of L1 inserts calculated for the High TRIM28 cohorts likely includes L1 inserts that occurred prior to upregulation of TRIM28 expression. Combined, we detect an agreement in the TRIM28 effect on cDNA synthesis/L1 insert length in three different experimental systems involving transiently transfected L1 and the WT or mutant full-length TRIM28 (or B box) or de novo inserts generated by endogenous L1s in the context of high or low levels of endogenous TRIM28 expression (Figures 7–9).

As some DNA repair genes, including genes in the NER pathway, have been revealed to suppress L1 retrotransposition, and some of these key genes are shown to be downregulated upon TRIM28 overexpression, our findings are consistent with the possibility that downregulation of DNA repair pathways could be a potential mechanism explaining the increase in L1 retrotransposition in this experimental system. In contrast however, we do not yet have a clear understanding of the mechanism by which TRIM28 interferes with the ORF2p-mediated cDNA synthesis. This is largely due to the very limited knowledge regarding TRIM28 oligomerization. However, it is worth discussing some possibilities. The phosphorylation status of TRIM28 has been recently reported to modulate Pol II pausing and transcriptional elongation at many mammalian genes (119). When phosphorylated at the Ser824 (an amino acid in Bromo domain), TRIM28 facilitates Pol II release from the pausing, resulting in rapid transcription of target genes, while S824 non-phosphorylated TRIM28 functions in stabilizing Pol II pausing (120–122). Even though the unphosphorylated TRIM28 status could be expected under overexpression conditions, the fact that the B box alone is sufficient to decrease cDNA length in the LEAP assay argues that the underlying mechanism here is independent of the S824 phosphorylation and, thus, likely different from the TRIM28 involvement with Pol II. However, the results of our step wise assessment of cDNA length in the LEAP assay are consistent with a ‘fall-off’ or ‘pausing’ of the ORF2p reverse transcriptase within a discrete region of the template instead of a gradual loss of processivity (Figures 7B, C and Supplementary Figure S6). Although a similar trend is not clearly observed with the length of the L1 inserts in cancer samples, this can be explained by the presence of L1 inserts generated prior to the increase in the TRIM28 expression in any given sample or a potential heterogeneity in TRIM28 expression in single cells.

Considering the interaction between ORF2p and TRIM28, we hypothesize that at least two possible non-mutually exclusive scenarios in how TRIM28 may affect ORF2p-dependent cDNA synthesis may exist. First, TRIM28 may directly or indirectly associate with ORF2p during initial stages of cDNA synthesis. Then, at some point it recruits a cellular factor(s) or binds to the RNA template which facilitates pausing or dissociation of the whole complex from the template RNA. Second, TRIM28 directly or indirectly binds to the mRNA template and interferes with ORF2p reverse transcription by blocking its forward progress, which ultimately leads to ORF2p pausing and/or dissociation from the RNA template. These models are consistent with the reported ability of TRIM28 to interact with both proteins and nucleic acids (123) and involvement of TRIM protein multimerization in enhancing its ability to suppress viral propagation (124). Our co-IP results in Supplementary Figure S5 support that the ORF2p interaction with TRIM28 is RNA-independent, potentially making the second scenario less favorable. However, we would like to remain cautious in ruling out RNA-dependent interaction because the RNA potentially mediating this interaction could be protected by the bound protein complex in this assay. Whether any of these models are plausible will be better understood once more information is gained about functional outcomes of TRIM28 multimerization. It will also be interesting to investigate if TRIM28 may have a similar effect on cDNA generated by viral reverse transcriptases or telomerase, which share significant sequence and structural similarity with the L1 RT (125,126).

Our findings of the effect of TRIM28 on L1 retrotransposition and cDNA synthesis in different cancer models may have important biological implications for understanding evolutionary arms race between the host and L1. Most of the 500 000 L1 elements in the human genome are 5′ truncated (25–27), but the mechanism(s) underlying this phenomenon that occurs during integration in the germline is unknown. A similar phenomenon exists in embryonic, normal adult, and transformed cells because the majority of tumor specific L1 inserts are also 5’ truncated (37,39,127). One explanation is that the L1 RT enzyme has limited processivity and often disengages from the L1 RNA template before synthesizing the full-length cDNA sequence, although this notion has been debated (128–131). It has also been reported that the length of L1 inserts varies with each L1 subfamily, with some polymorphism outside of the RT domain being proposed to play a role in the ORF2p ability to bind its RNA (33). Here we report a cellular factor that modulates cDNA lengths and the length of L1 inserts in cultured cells and seemingly *in vivo*. TRIM28 is ubiquitously expressed throughout development and in adult tissues. In embryonic stem cells the expression level of L1 and TRIM28 are both high (50,55,132). It is possible that TRIM28 initially evolved to suppress generation of new full-length L1 inserts in the germline to prevent L1’s evolutionary success. However, L1 may have adapted to capitalize on the very same mechanism to facilitate its mobilization. This may be the scenario we observe in cancer cells where increasing tumor heterogeneity due to *de novo* L1 integration is beneficial to driving cancer evolution regardless of the size of L1 inserts. The reverse order of these evolutionary steps is also possible. According to this hypothesis, L1 may have evolved to use TRIM28 to increase the number of its inserts. To counteract this the host utilized the same mechanism to reduce the number of full-length L1 inserts to halt L1 propagation.

In summary, we have identified TRIM28 as a suppressor of ORF2p-mediated cDNA synthesis, but overall, a positive regulator of L1 retrotransposition. Both effects rely on the B Box facilitated oligomerization. These findings have evolutionary and disease relevant consequences that may be related to other viral and non-viral RT containing proteins. Although TRIM28 is rarely mutated in human cancers, its expression is often deregulated with overexpression being linked to poor survival. Our findings support that the ability of TRIM28 to promote tumorigenesis may include its potential to increase L1-induced genomic instability. Future mechanistic studies of TRIM and L1 proteins will be needed to provide a more comprehensive understanding of functional outcomes of their interactions on genome stability, evolution, and cancer pathogenesis.

## DATA AVAILABILITY

The raw data files for RNA-seq analysis in this manuscript are available under BioProject: PRJNA925462.

## Supplementary Material

gkad247_Supplemental_Files
