# Supplementary material for: A novel role of TRIM28 B box domain in L1 retrotransposition and ORF2p-mediated cDNA synthesis
Source: Nucleic Acids Res. 2023 Apr 18;51(9):4429–50. doi: 10.1093/nar/gkad247 (PMC10201437; doi:10.1093/nar/gkad247)
Supplement: gkad247_Supplemental_Files [file gkad247_supplemental_files.zip › Final-Du et al Supplementary Figure.docx]

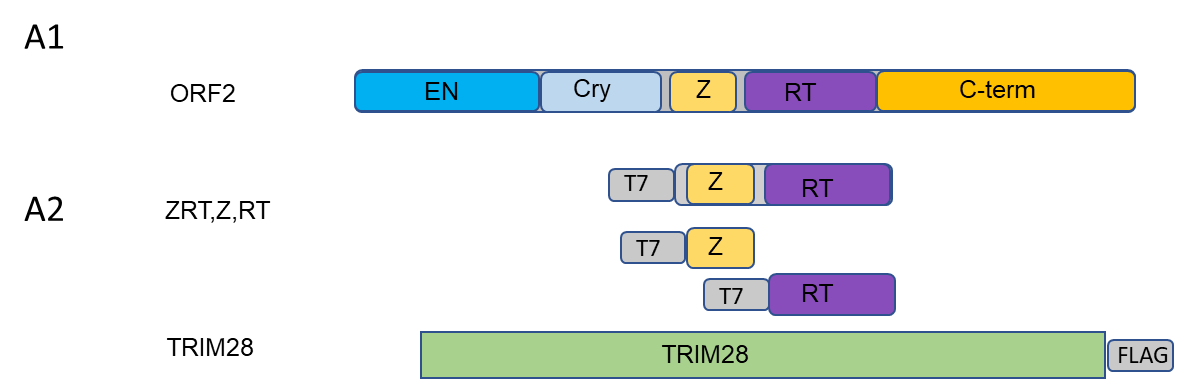

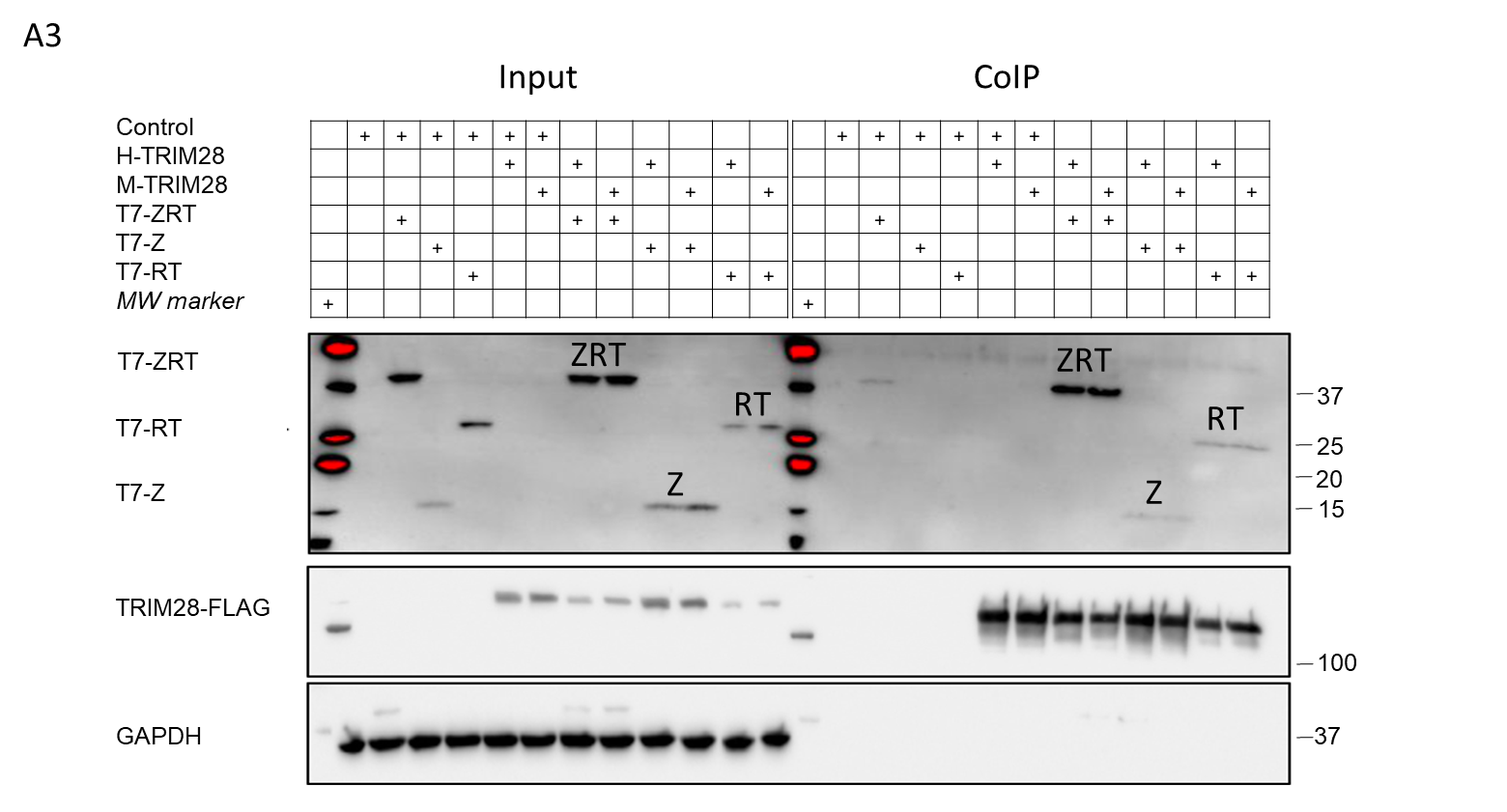

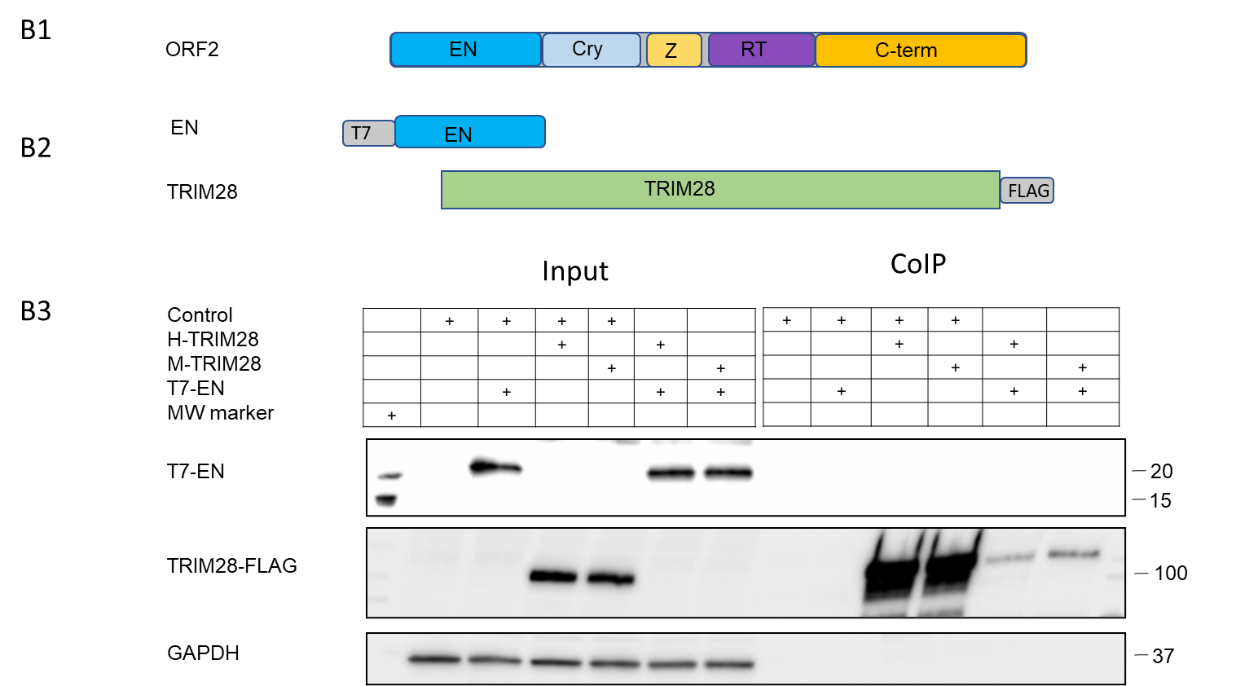


**Supplementary Figure 1. Multiple domains of L1 ORF2p interact with TRIM28**


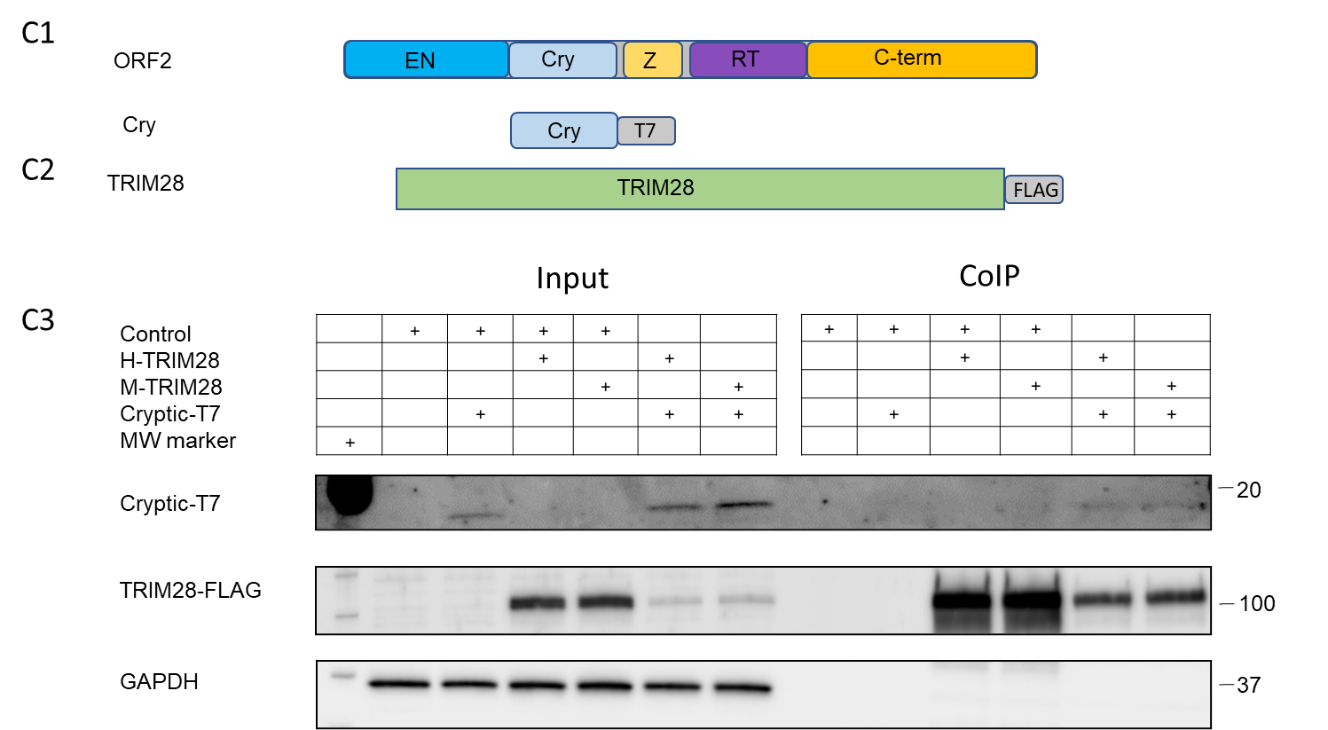


**
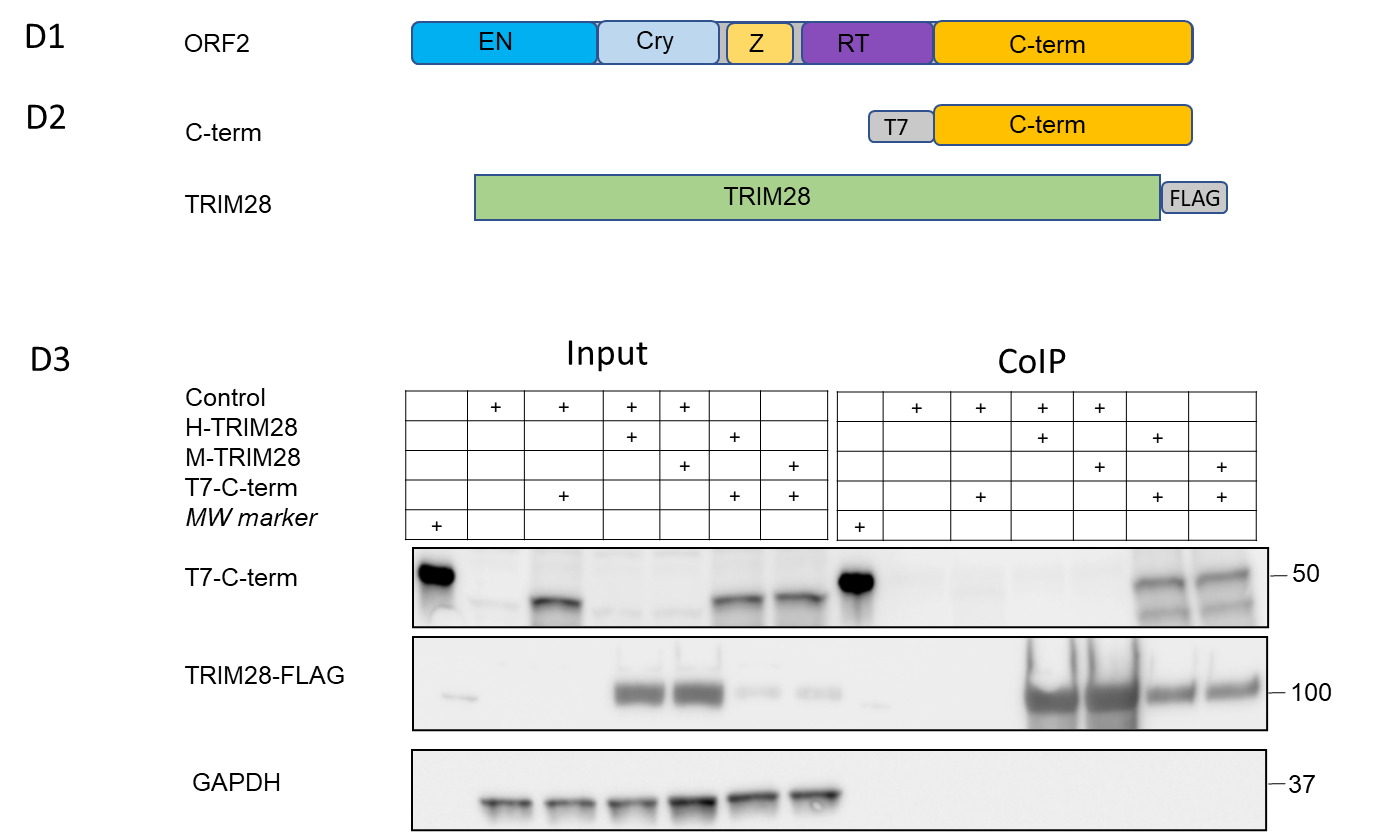
**

**Supplementary Figure 1**. **Multiple domains of ORF2p interact with TRIM28**. **(A1)** Schematic of full-length ORF2p. **(A2)** Schematic of expression plasmids used for co-transfection in HeLa cells followed by co-Immunoprecipitation and western blot assays. **(A3)** Western blot analysis using T7 or FLAG-specific Abs. T7 tagged ZRT, Z or RT expression plasmids are individually co-expressed with FLAG tagged Human or Mouse TRIM28 expression plasmids in HeLa cells. FLAG antibody coupled beads are used to pull down FLAG-tagged proteins. The recovered protein complexes are subjected to western blot analysis. GAPDH is used as loading control. Input corresponds to assessment of protein expression in whole cell lysates. CoIP corresponds to the assessment of co-IP results. **(B1)** Schematic of full-length ORF2p. **(B2)** Schematic of plasmids used for co-Immunoprecipitation assay in HeLa cells. **(B3)** Western blot analysis using T7 or FLAG-specific Abs. T7 tagged EN is co-expressed with FLAG-tagged Human or Mouse TRIM28 in HeLa cells. FLAG antibody coupled beads are used to pull down FLAG-tagged proteins. The recovered protein complexes are subjected to western blot analysis. GAPDH is used as loading control. Input corresponds to assessment of protein expression in whole cell lysates. CoIP corresponds to the assessment of co-IP results. Note a significant reduction in the TRIM28 expression levels when co-transfected with an EN-expression plasmid potentially due to its ability to cut transfected DNA. **(C1)** Schematic of full-length ORF2p. **(C2)** Schematic of plasmids used for co-Immunoprecipitation assay in HeLa cells. **(C3)** Western blot analysis using T7 or FLAG-specific Abs. T7 tagged Cryptic is co-expressed with FLAG tagged Human or Mouse TRIM28 in HeLa cells. FLAG antibody coupled beads are used to pull down. The recovered protein complexes are subjected to western blot analysis. GAPDH is used as loading control. Input corresponds to assessment of protein expression in whole cell lysates. CoIP corresponds to the assessment of co-IP results. **(D1)** Schematic of full-length ORF2p. **(D2)** Schematic of plasmids used for co-Immunoprecipitation assay in HeLa cells. **(D3)** Western blot analysis using T7 or FLAG-specific Abs. T7 tagged C-term is co-expressed with FLAG tagged Human or Mouse TRIM28 in HeLa cells. FLAG antibody coupled beads are used to pull down. The recovered protein complexes are subjected to western blot analysis. GAPDH is used as loading control. Input corresponds to assessment of protein expression in whole cell lysates. CoIP corresponds to the assessment of co-IP results.

**Supplementary Figure 2**. **Multiple domains of TRIM28 interact with ORF2 ZRT fragment**

A1


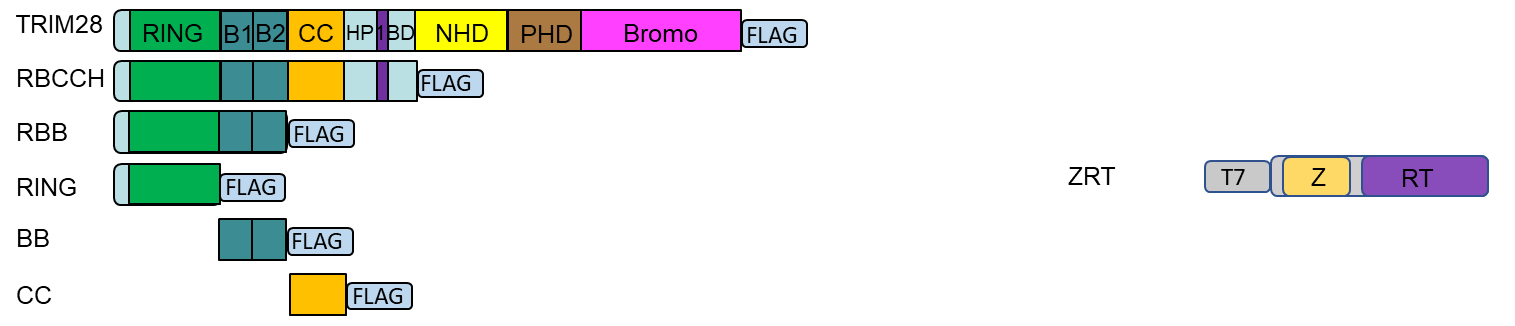


A2


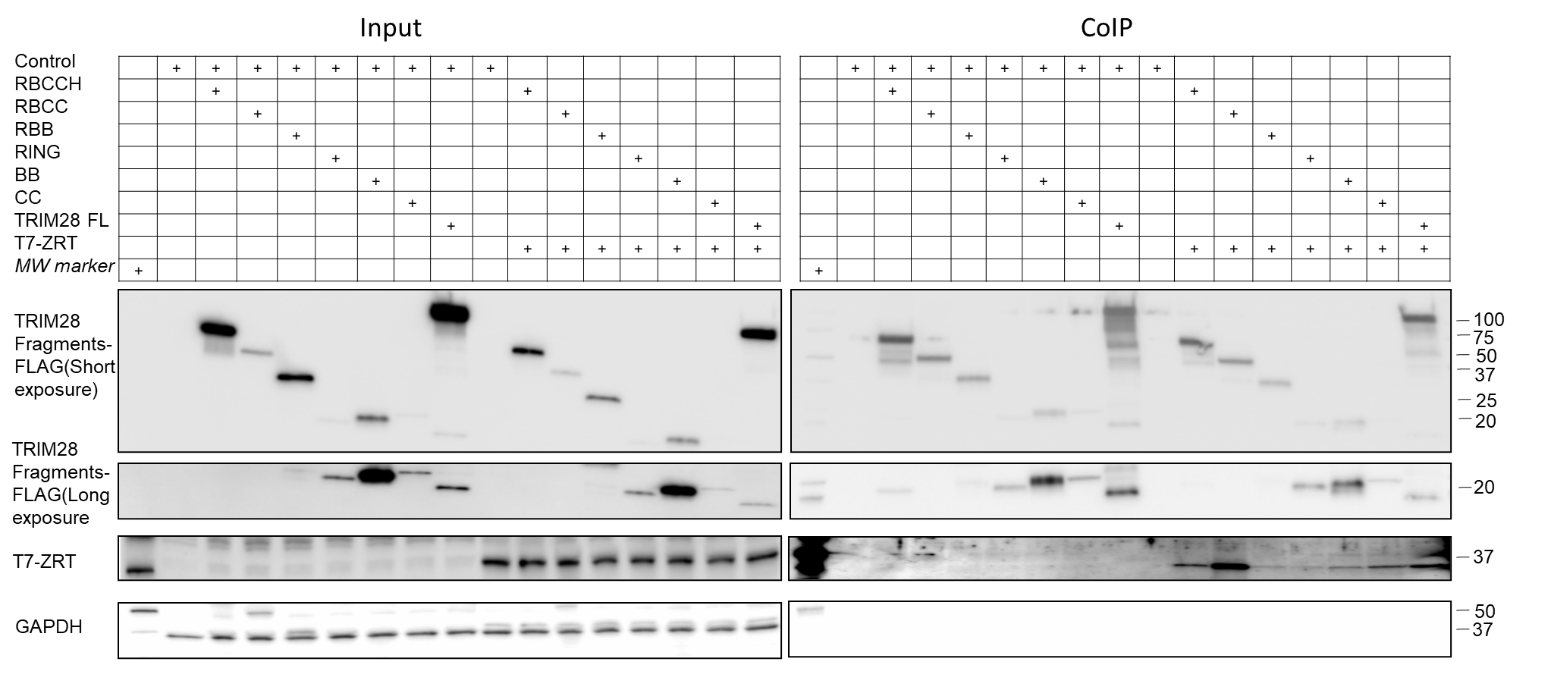


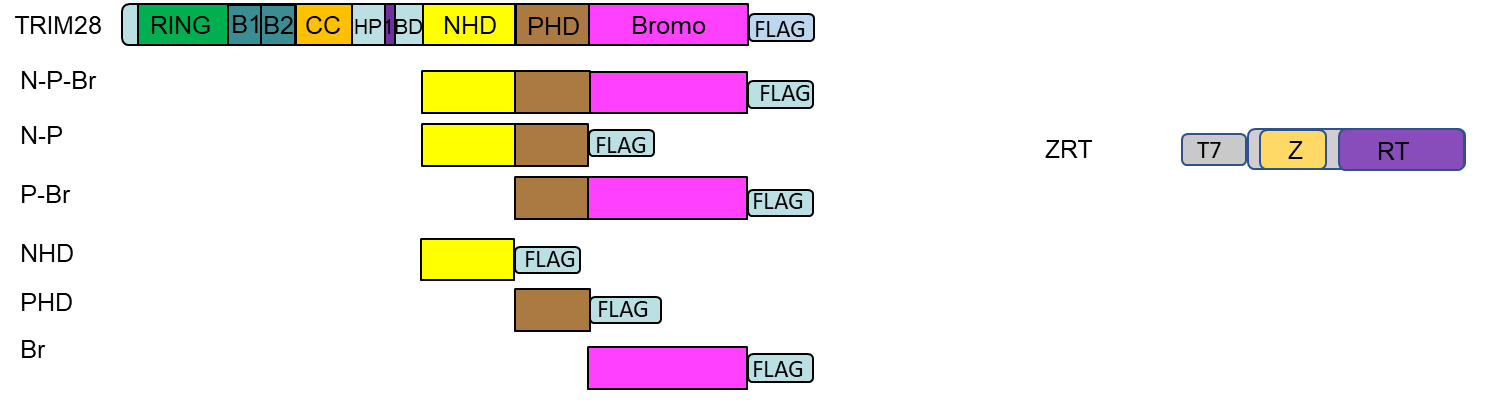
 **
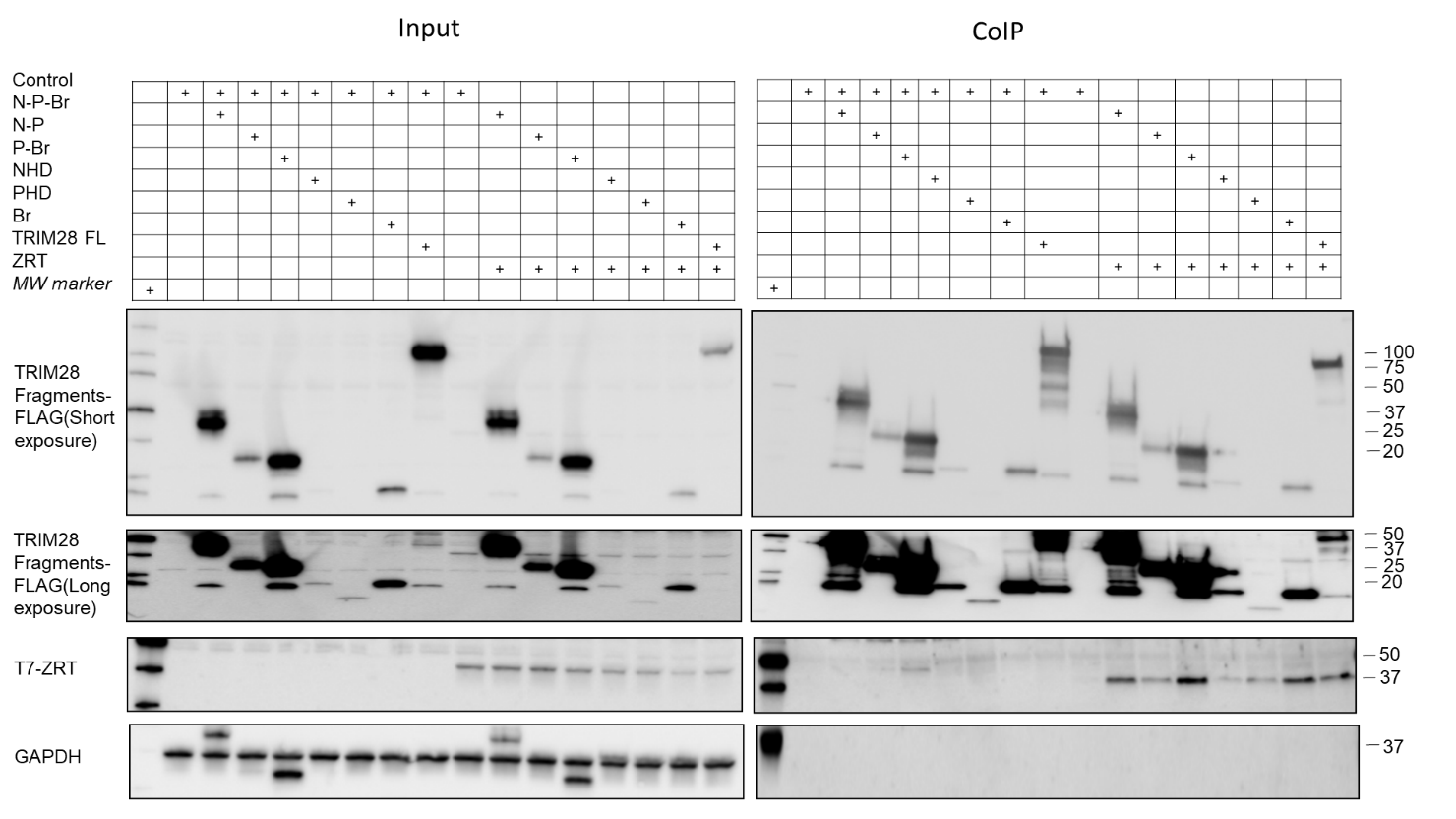
**

B2

B1

**Supplementary Figure 2**. **Multiple domains of TRIM28 interact with ORF2 ZRT fragment. (A1)** Schematic of expression plasmids used for co-transfection and co-Immunoprecipitation assays in HeLa cells. Left: full-length TRIM28 and N-terminal TRIM28 fragments, Right: ZRT fragment of ORF2p.  **(A2)** Results of western blot analysis using T7 or FLAG-specific Ab. Individual FLAG tagged N-terminal H-TRIM28 fragments or full-length H-TRIM28 are co-expressed with T7 tagged ZRT fragment in HeLa cells. FLAG antibody coupled beads are used to pull down FLAG-tagged proteins. The recovered protein complexes are subjected to western blot analysis. GAPDH is used as loading control. Input corresponds to assessment of protein expression in whole cell lysates. CoIP corresponds to the assessment of co-IP results. **(B1)** Schematic plasmids used for co-Immunoprecipitation assay in HeLa cells. Left: full-length TRIM28 and C-terminal TRIM28 fragments, Right: ZRT fragment from ORF2p. **(B2)** FLAG tagged C-terminal H-TRIM28 fragment or full-length H-TRIM28 is co-expressed with T7 tagged ZRT fragment in HeLa cells. FLAG antibody coupled beads are used to pull down cell lysates. The precipitated protein complexes are subjected to western blot analysis. GAPDH is used as loading control. Input corresponds to assessment of protein expression in whole cell lysates. CoIP corresponds to the assessment of co-IP results.

A1

**Supplementary Figure 3. TRIM28 and TRIM28 fragments colocalize with ORF2p**

A2


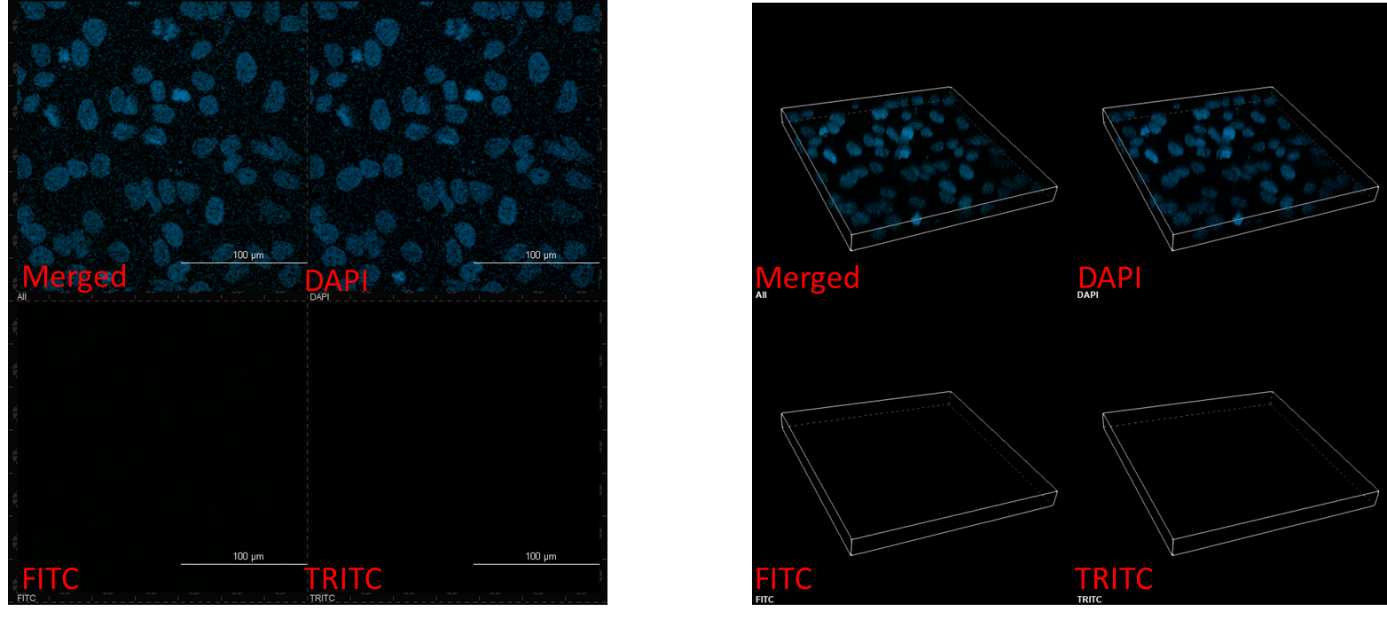


B1 B2


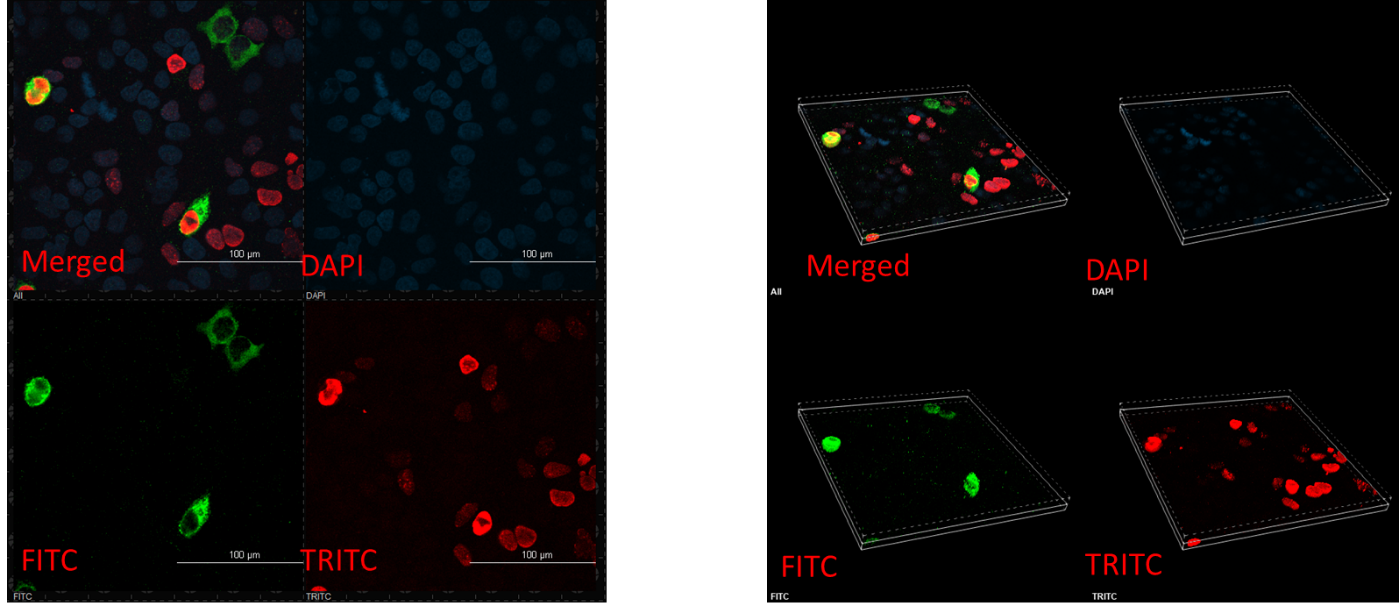


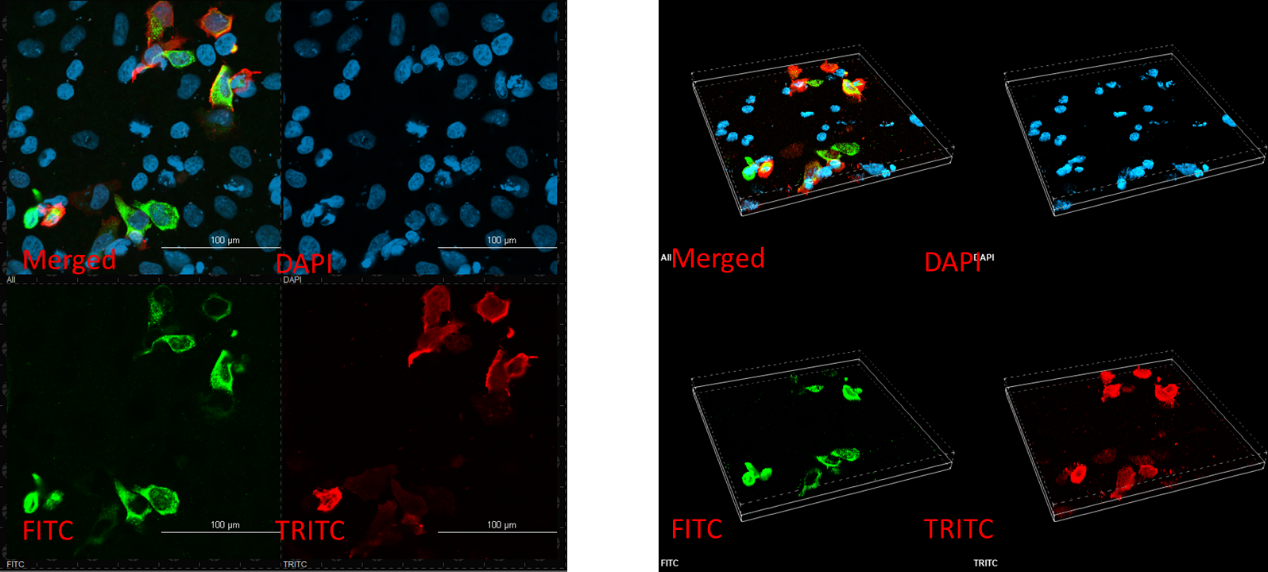


C1 C2

D1 D2


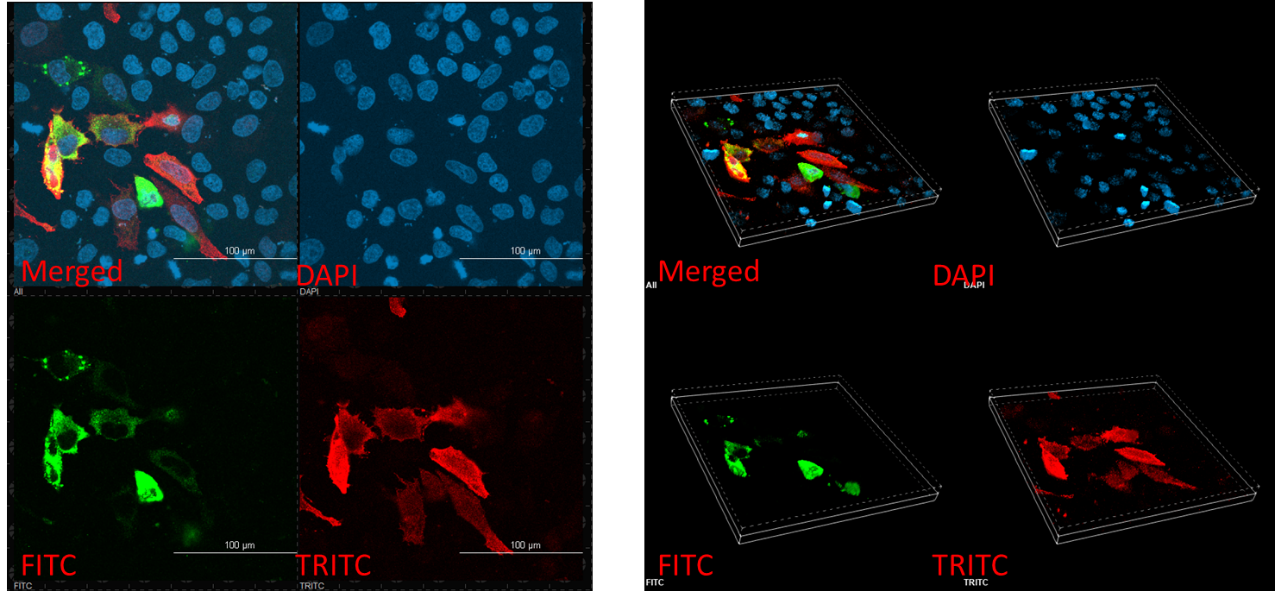


E1 E2
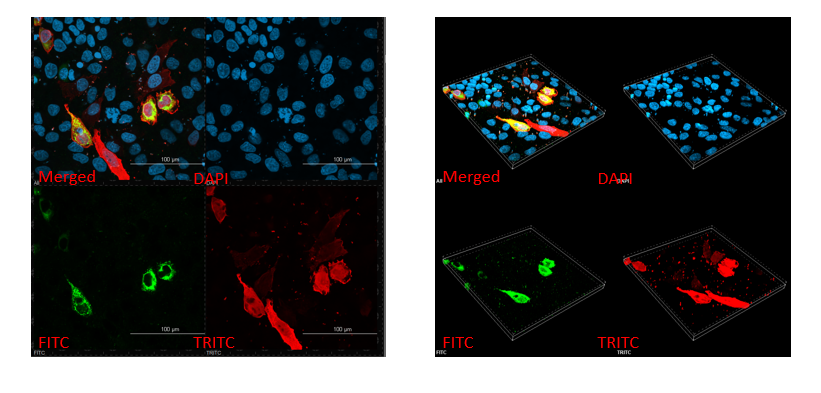


F1 F2


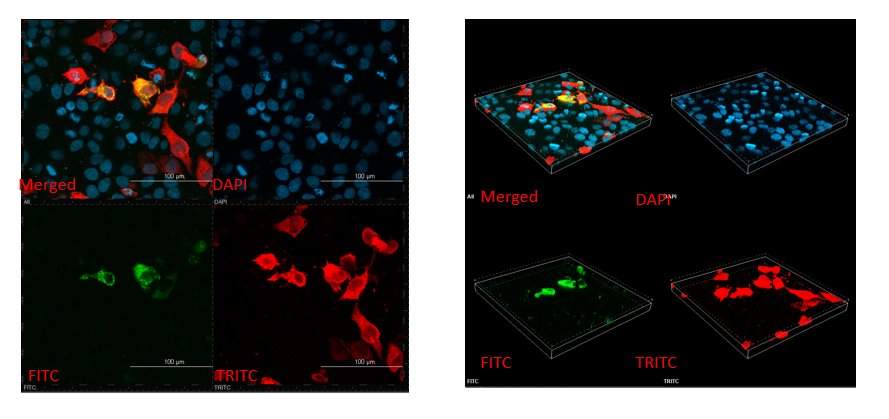


G1 G2


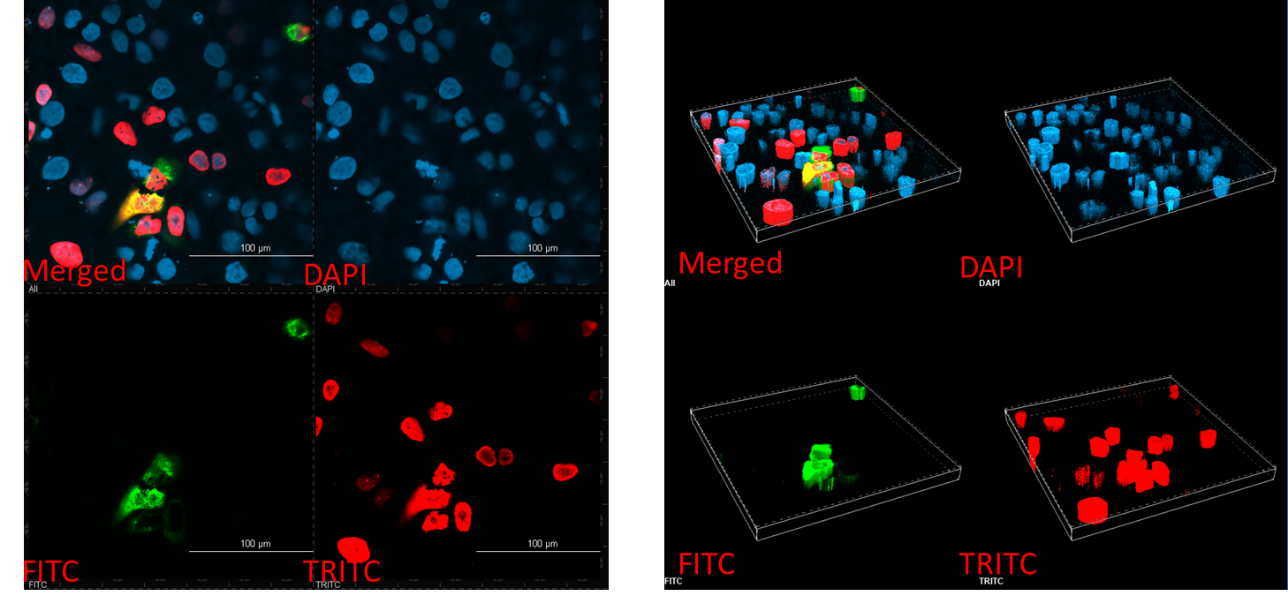


**Supplementary Figure 3. ORF2p and TRIM28 interact in both the nucleus and in the cytoplasm.** Representative confocal microscopy images of the colocalization of full-length or fragmented TRIM28 (red TRITC stain) and ORF2p (green FITC stain) were obtained using HeLa cells transfected with empty vector PCDNA 3.1+ or TRIM28 fragments and ORF2p. **(A1-A2)**. Horizontal views (x-y sections) and lateral views (z-x sections) of cells are shown with DAPI nuclei (blue), respectively, for HeLa cells transfected with control vector PCDNA 3.1+. **(B1-B2).** Horizontal views (x-y sections) and lateral views (z-x sections) of cells are shown with DAPI nuclei (blue), respectively, for HeLa cells transfected with RBCCH (TRITC red) +ORF2 (FITC green) **(C1-C2)** Horizontal views (x-y sections) and lateral views (z-x sections) of cells are shown with DAPI nuclei (blue), respectively, for HeLa cells transfected with RBB (TRITC red) +ORF2(FITC green) **(D1-D2)** Horizontal views (x-y sections) and lateral views (z-x sections) of cells are shown with DAPI nuclei (blue), respectively, for HeLa cells transfected with BB (TRITC red) +ORF2 (FITC green) (**E1-E2)** Horizontal views (x-y sections) and lateral views (z-x sections) of cells are shown with DAPI nuclei (blue), respectively, for HeLa cells transfected with NPBr (TRITC red) +ORF2 (FITC green) **(F1-F2)** Horizontal views (x-y sections) and lateral views (z-x sections) of cells are shown with DAPI nuclei (blue), respectively, for HeLa cells transfected with Br (TRITC red) +ORF2 (FITC green) **(G1-G2)** Horizontal views (x-y sections) and lateral views (z-x sections) of cells are shown with DAPI nuclei (blue), respectively, for HeLa cells transfected with FL-TRIM28 (TRITC red) +ORF2 (FITC green).

**Supplementary Figure 4. ORF2 ZRT fragment and TRIM28 interact in both the nucleus and in the cytoplasm**


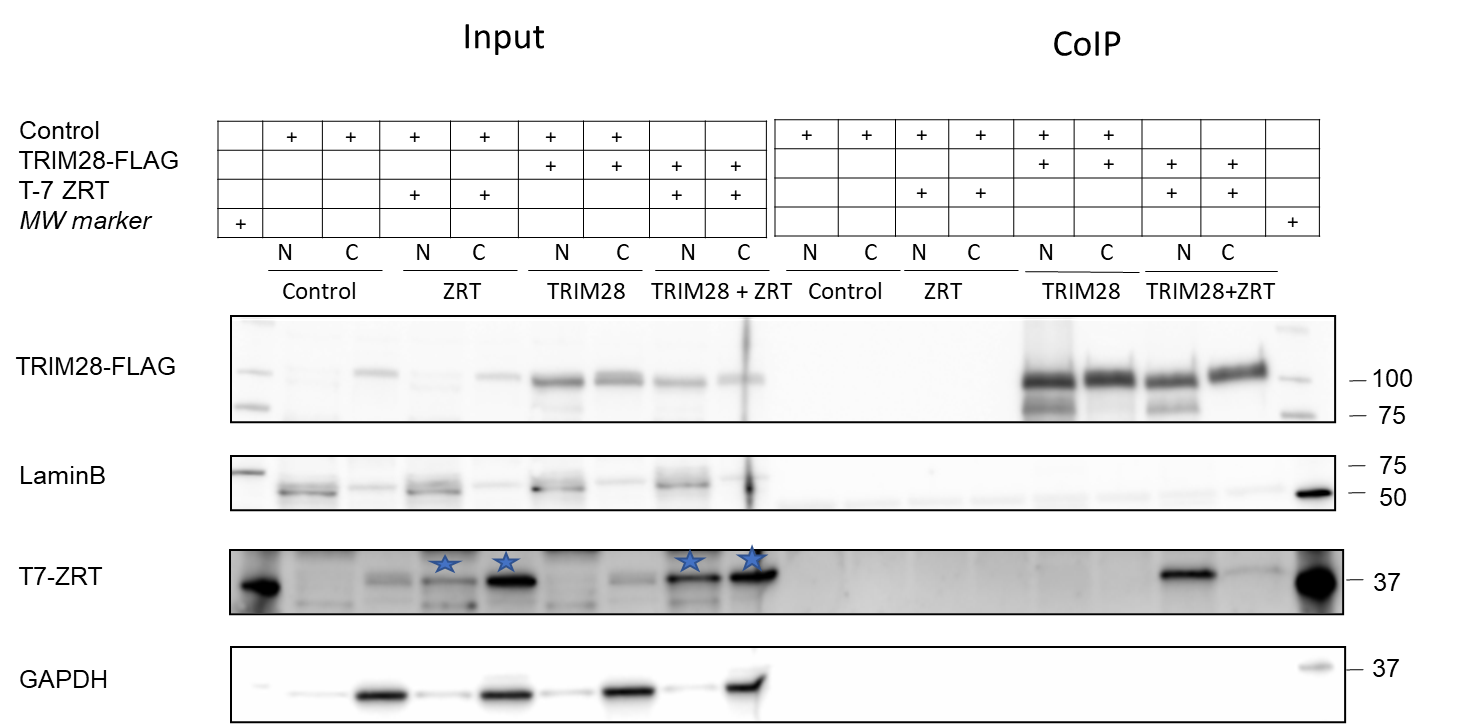


**Supplementary Figure 4. ORF2 ZRT fragment and TRIM28 interact in both the nucleus and in the cytoplasm.** FLAG tagged TRIM28 and T7 tagged ZRT are co-expressed in HeLa cells. Co-immunoprecipitation and western blot analysis are performed with nuclear (N) and cytoplasmic (C) fractions. GAPDH and Lamin B are used as loading controls. Presence of T7 ZRT indicates an interaction. Control lane indicates cells transfected with an empty plasmid.Stars indicate the T7-ZRT

**Supplementary Figure 5. ORF2p-TRIM28 interaction is maintained upon RNase A treatment**


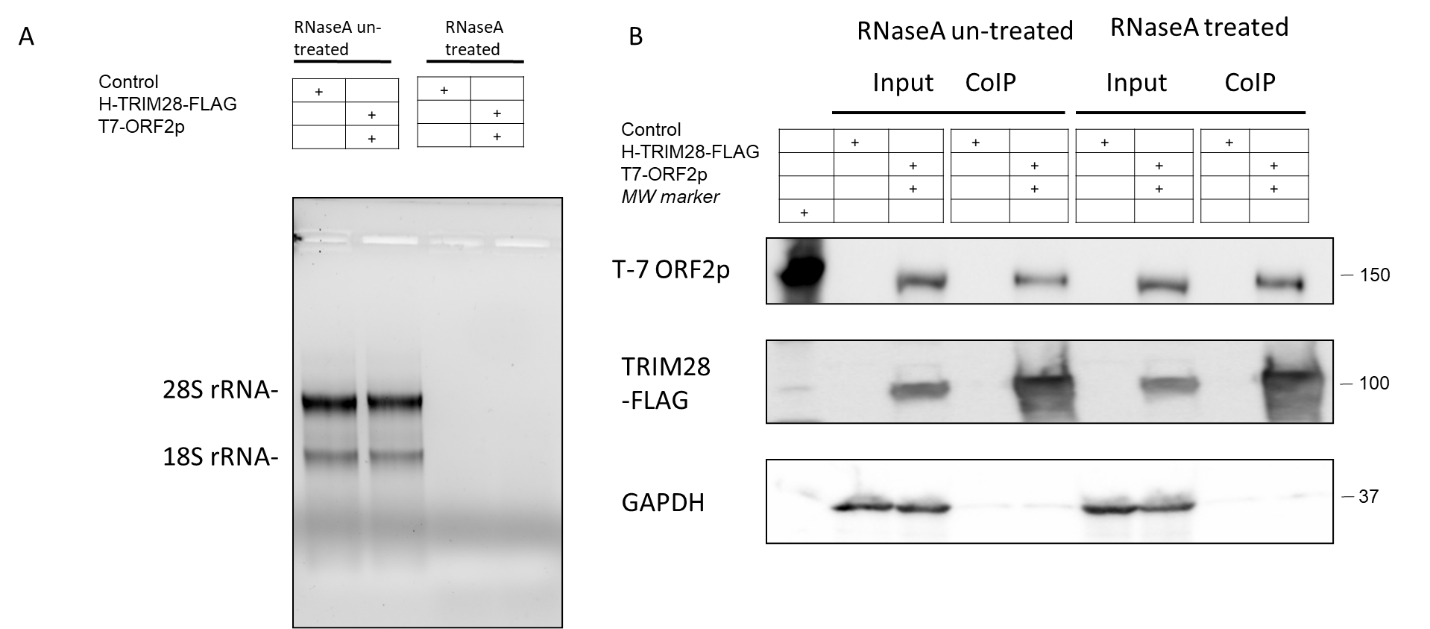


**Supplementary Figure 5. ORF2p-TRIM28 interaction is maintained upon RNAse treatment**

Transient co-transfection in HeLa cells was performed with either empty vector pCDNA 3.1+ or TRIM28-FLAG and T7-ORF2p. Cell lysates were either treated (+) or not treated (−) with 5µl RNase A for 20 min at 25°C. (A) Cytoplasmic RNA was extracted and prepared as previously described (102). RNA analysis by agarose gel electrophoresis. The 28S and 18S RNA bands are indicated. (B) Co-immunoprecipitation and western blot analysis were performed with indicated samples. GAPDH was used as loading control. Presence of T7 ORF2p indicates an interaction. Control lane indicates cells transfected with an empty plasmid PCDNA 3.1+. FLAG antibody coupled beads are used to pull down cell lysates.

**Supplementary Figure 6. Mutant TRIM28 or BB domain retains interaction with ORF2p.**

A1


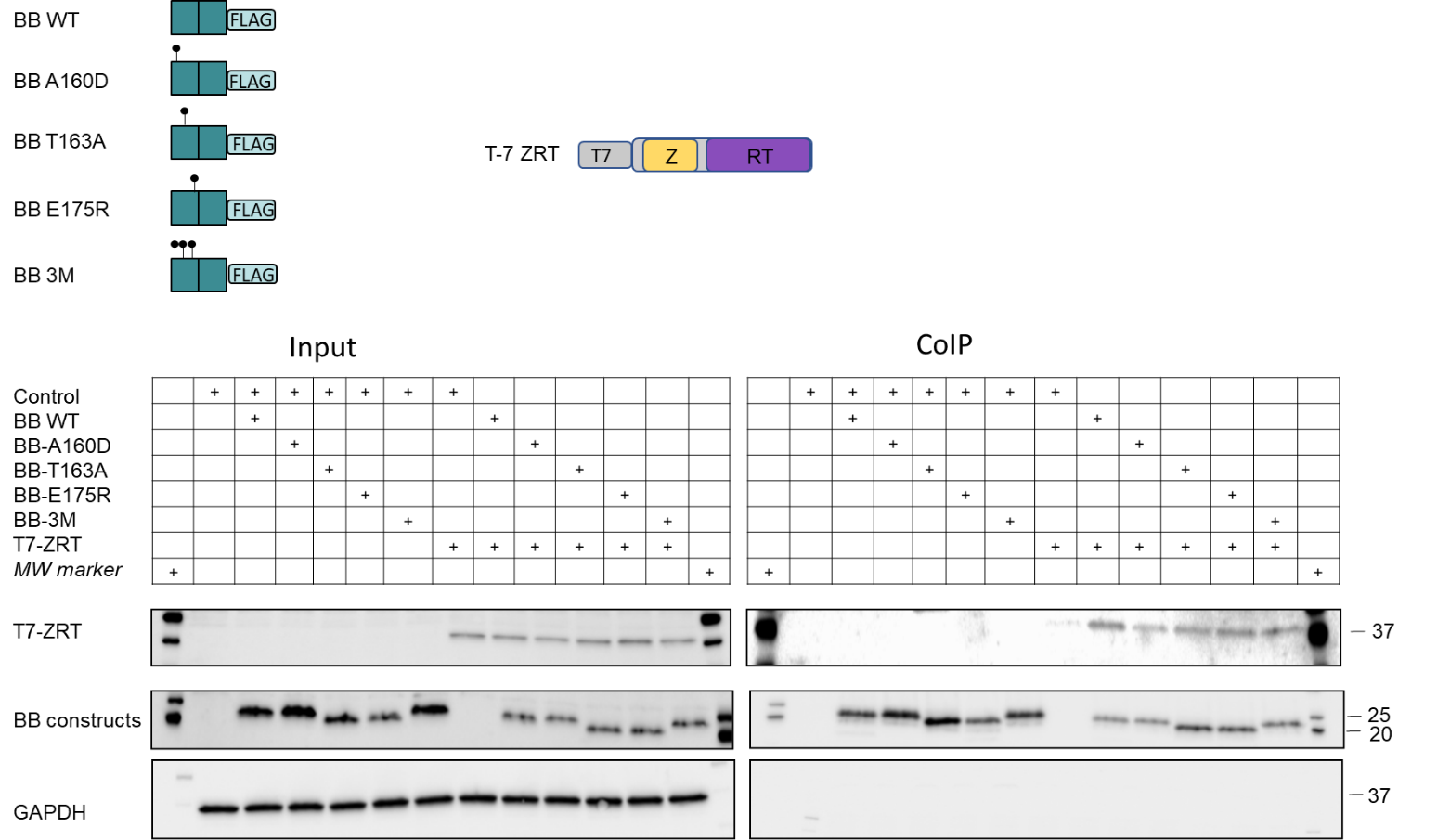


A2


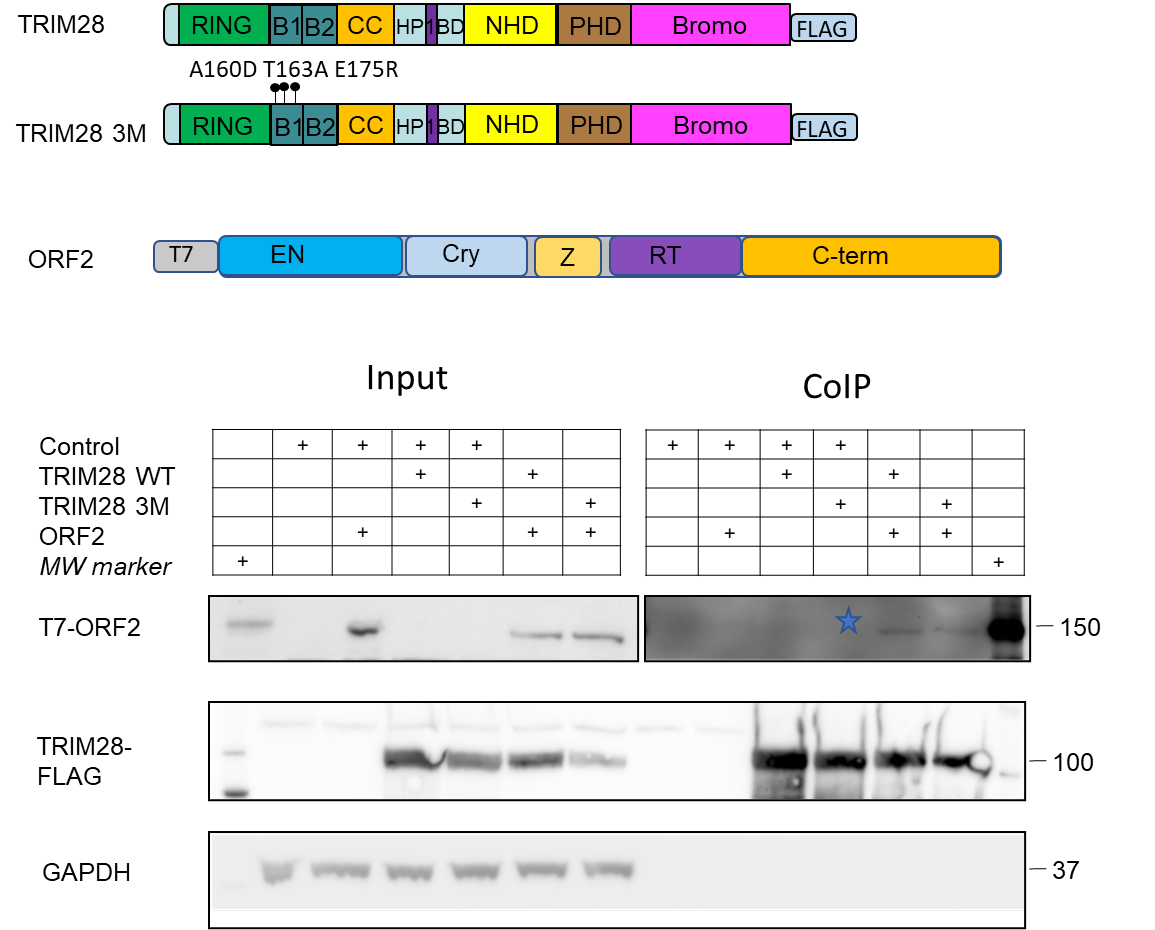


B2

B1


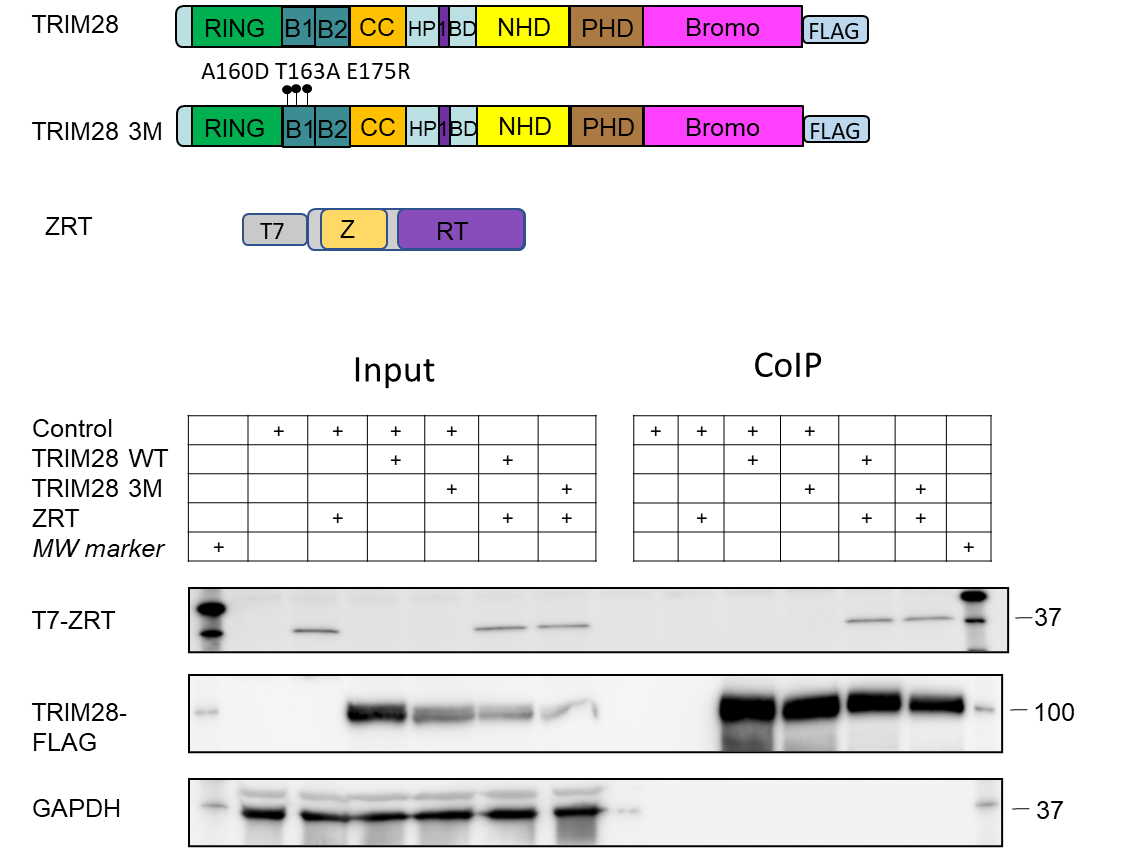


C1

C2


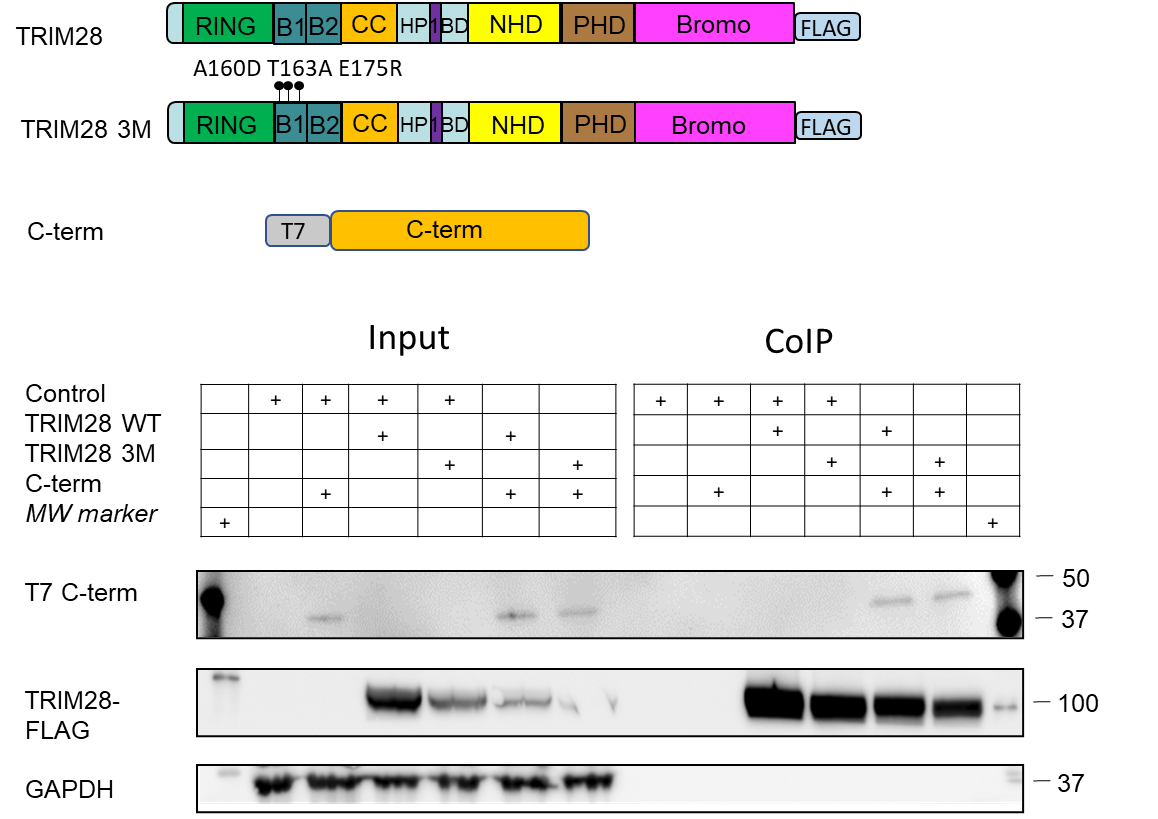


D1

D2

**Supplementary Figure 6**. **Mutant TRIM28 or BB domain retains interaction with ORF2p.** **(A1)** Schematic of TRIM28 B box variants (left) and ZRT from ORF2p (right). BB is B box, WT is wild type, single or triple mutations are indicated using single letter amino acid code and aa position in the human wt TRIM28 protein. **(A2)** Indicated FLAG tagged BB variant is co-expressed with T7 tagged ZRT in HeLa cells. FLAG antibody coupled beads are used to pull down cell lysates. The precipitated protein complexes are subjected to western blot analysis. GAPDH is used as loading control. Input corresponds to assessment of protein expression in whole cell lysates. CoIP corresponds to the assessment of co-IP results. Presence of T7-ZRT on the CoIP side indicates an interaction. **(B1)** Schematic of TRIM28 variants (top) and ORF2p (bottom). **(B2)** FLAG tagged TRIM28 WT or TRIM28 3M is co-expressed with T7 tagged ORF2p in HeLa cells. FLAG antibody coupled beads are used to pull down cell lysates. The precipitated protein complexes are subjected to western blot analysis. Presence of T7-ORF2 (indicated by blue star) on the CoIP side indicates an interaction. **(C1)** Schematic of TRIM28 variants (top) and ZRT from ORF2p (bottom). **(C2)** FLAG tagged TRIM28 WT or TRIM28 3M is co-expressed with T7 tagged ZRT in HeLa cells. FLAG antibody coupled beads are used to pull down cell lysates. The precipitated protein complexes are subjected to western blot analysis. Presence of T7 ZRT on the CoIP side indicates an interaction. **(D1)** Schematic of TRIM28 variants (top) and C-term from ORF2p (bottom). **(D2)** FLAG tagged TRIM28 WT or TRIM28 3M is co-expressed with T7 tagged C-term fragment in HeLa cells. FLAG antibody coupled beads are used to pull-down cell lysates. The precipitated protein complexes are subjected to western blot analysis. Presence of T7 C-term on the CoIP side indicates an interaction.

**Supplementary Figure 7. TRIM28 WT and BB WT increase L1 Retrotransposition in U2OS cells whereas TRIM28 3M and BB 3M lose this ability**


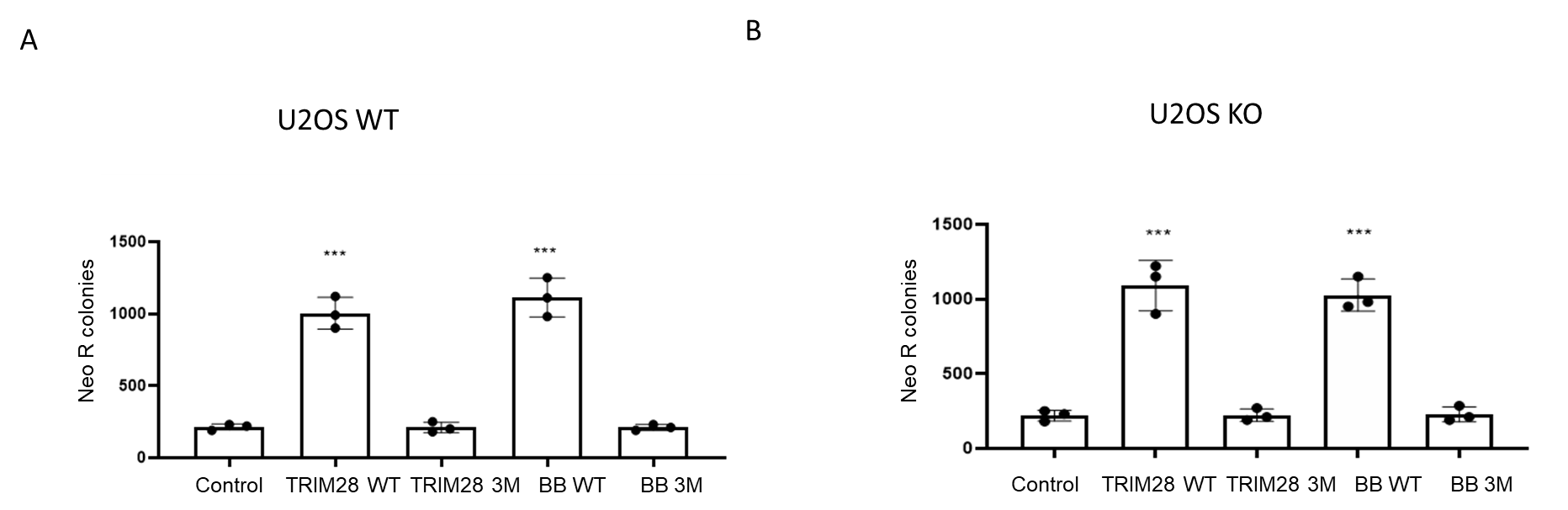


**Supplementary Figure 7**. **TRIM28 WT and BB WT increase L1 Retrotransposition in U2OS cells whereas TRIM28 3M and BB 3M lose this ability.** **(A)** L1 retrotransposition result. Wild type U2OS cells (U2OS WT) are transiently co-transfected with plasmids expressing human neomycin tagged L1 (L1Neo) and indicated full-length TRIM28 or BB variants. The number of G418(Neo) resistant colonies is counted after 14-days selection. Asterisks (*) denote statistical significance between listed constructs and the control (n = 3, t-test, ***P < 0.001). Dots represent the number of Neo R colonies observed in individual experiments. **(B)** L1 retrotransposition result. U2OS TRIM28 KO cells are transiently co-transfected with plasmids expressing human neomycin tagged L1 (L1Neo) and indicated full-length TRIM28 or BB variants. The number of G418(Neo) resistant colonies is counted after 14-days selection. Asterisks (*) denote statistical significance between listed constructs and the control (n = 3, t-test, ***P < 0.001). Dots represent number of Neo R colonies observed in individual experiments. Error bars represent the standard deviation (SD).

**Supplementary Figure 8. TRIM28 overexpression does not change L1Neo ORF1p expression levels**


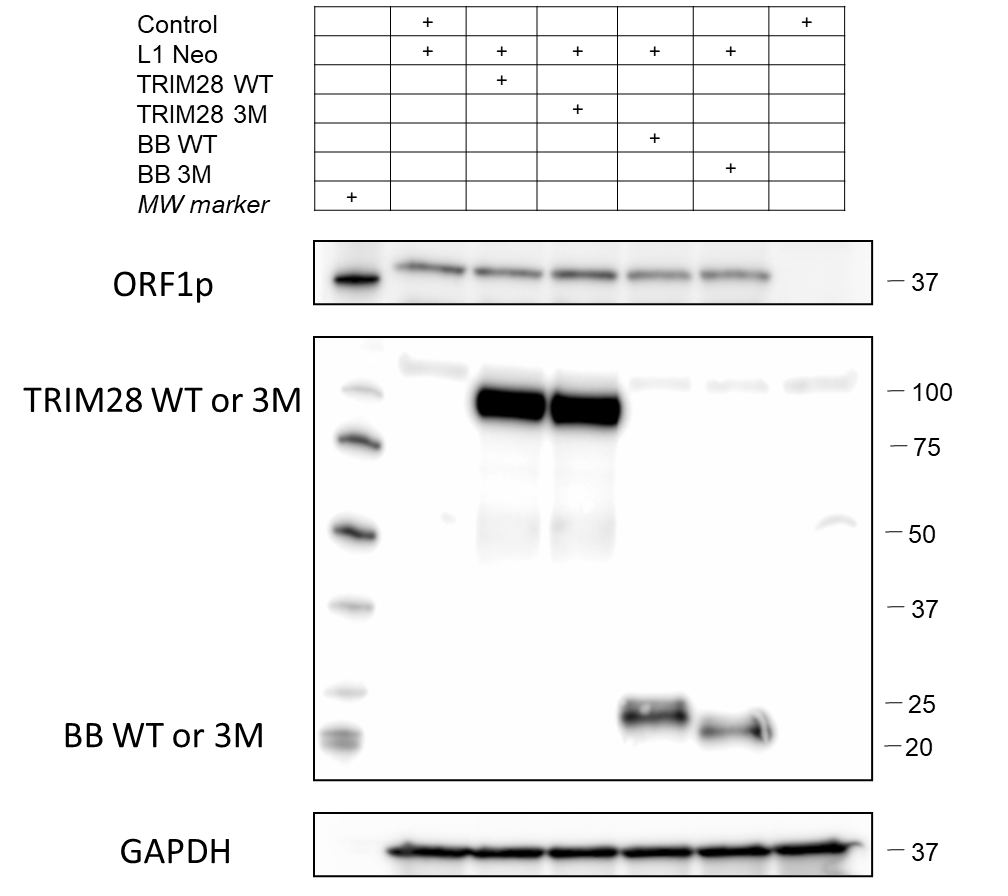


**Supplementary Figure 8. TRIM28 overexpression does not change L1Neo ORF1p expression levels**

HeLa cells were transiently co-transfected with plasmids expressing human neomycin tagged L1 (L1Neo) and indicated full-length TRIM28 or BB variants. The cell lysates were harvested 48h post-transfection and subjected to western blot analysis. ORF1 was detected using anti-ORF1 antibodies. TRIM28 or BB variants were detected using anti-FLAG antibodies. GAPDH was used as loading control (the lower bands).

**Supplementary Figure 9. TRIM28 WT and BB WT increase L1 Blast Retrotransposition**

**
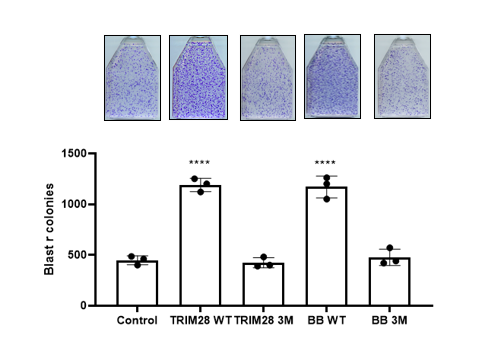
**

**Supplementary Figure 9. TRIM28 WT and BB WT increase L1 Blast Retrotransposition**

L1 retrotransposition result. HeLa cells were transiently co-transfected with plasmids expressing Blasticidine tagged L1 (L1 Blast) and indicated full-length TRIM28 or BB variants. The number of Blasticidine resistant colonies was counted after 14-days of selection. Asterisks (*) denote statistical significance between listed constructs and the control (n = 3, t-test, ***P < 0.001). Dots represented the number of Blasticidine colonies observed in individual experiments. Error bars represent the standard deviation (SD).

**Supplementary Figure 10. DNA repair pathway is down-regulated upon TRIM28 WT overexpression**


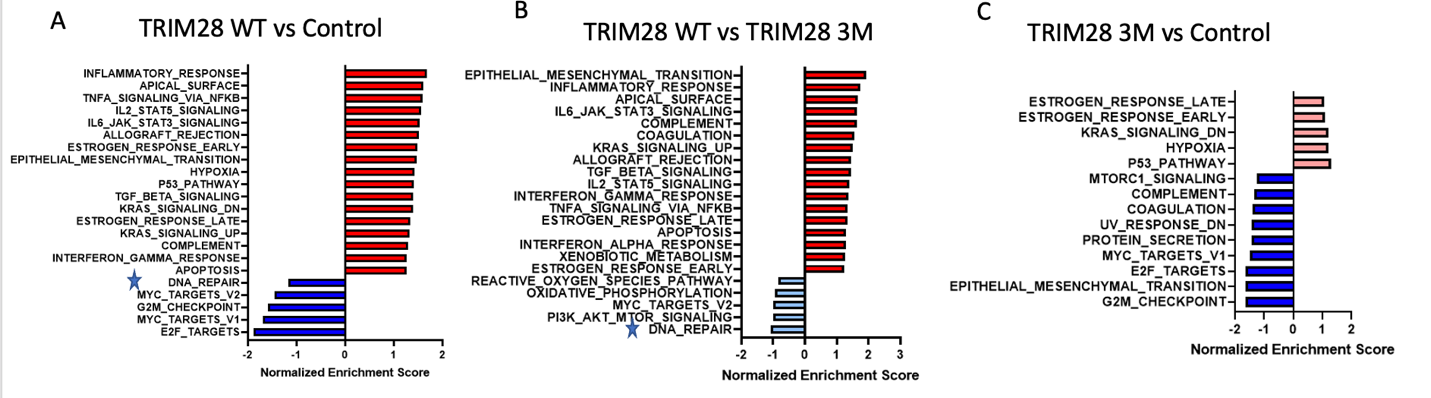


**Supplementary Figure 10. Differentially regulated pathways in HeLa cells over-expressing control plasmid, TRIM28 WT, or TRIM28 3M. (A)** Gene set enrichment analysis (GSEA) shows the normalized enrichment score (NES) for the differentially expressed gene sets in TRIM28 WT sample versus control. Star points out the DNA repair pathway. **(B)** Gene set enrichment analysis (GSEA) shows the normalized enrichment score (NES) for the differentially expressed gene sets in TRIM28 WT versus TRIM28 3M. Star points out the DNA repair pathway. **(C)** Gene set enrichment analysis (GSEA) shows the normalized enrichment score (NES) for the differentially expressed gene sets in TRIM28 3M versus Control.

**Supplementary Figure 11. Overexpression of TRIM28 WT or TRIM28 3M does not alter the expression of endogenous L1 mRNA or the profile of expressed L1 Loci in HeLa cells**


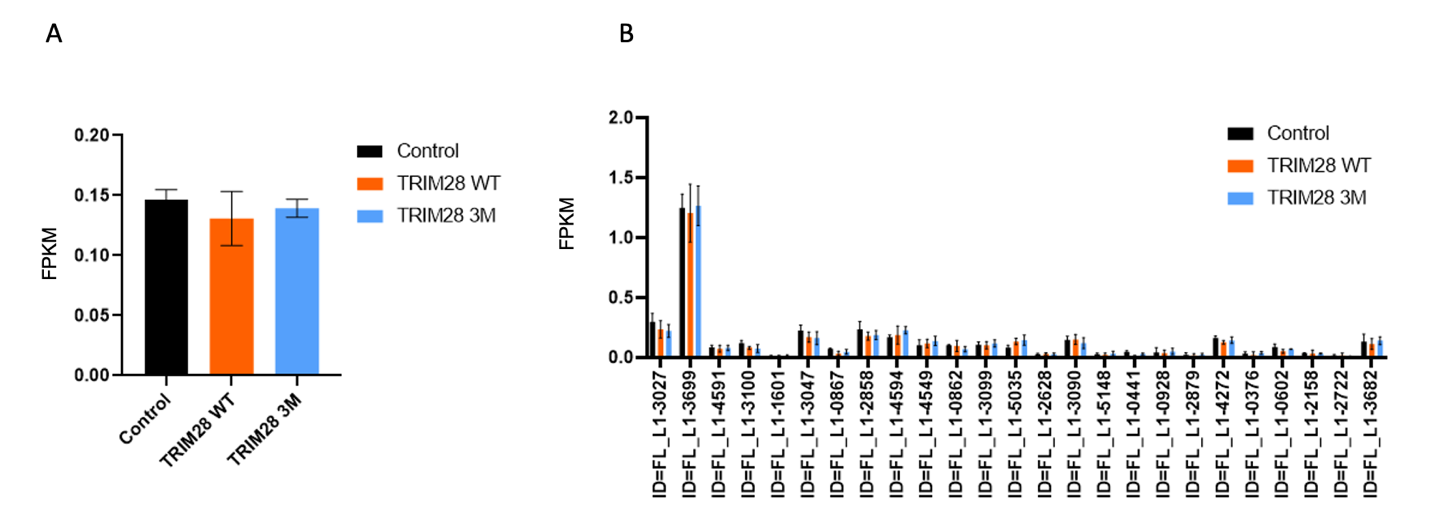


**Supplementary Figure 11. Overexpression of TRIM28 WT or TRIM28 3M does not alter the expression of endogenous L1 mRNA or the profile of expressed L1 Loci in HeLa cells**

RNA-seq analysis was performed to analyze the expression of endogenous L1 in HeLa cells over-expressing control plasmid, TRIM28 WT, or TRIM28 3M. Analysis was performed with 4 replicates for each treatment. **(A)** The bar graph shows the expression level (FPKM) of total L1 expression in each sample, i.e. the sum of FPKM of all L1 loci identified as expressed in that sample; Error bars indicate standard deviation. Unpaired two sample t test was performed to compare TRIM28 WT vs. control (P= 0.2463); TRIM28 3M vs. control (P=0.4983) **(B)** Number of reads corresponding to individually expressed L1 Loci in each of the indicated samples are plotted to assess their expression status upon overexpression of WT or 3M TRIM28 in HeLa cells. Error bars represent the standard deviation (SD).


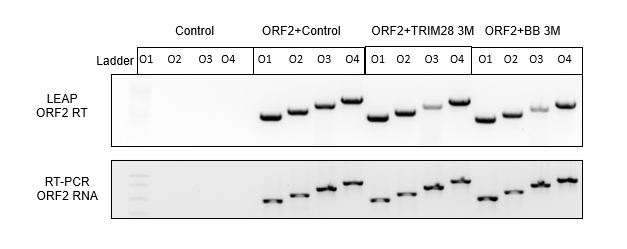


**Supplementary Figure 12. TRIM28 3M and BB 3M do not affect ORF2p-dependent cDNA synthesis**

**Supplementary Figure 12.** **TRIM28 3M and BB 3M do not affect ORF2p-dependent cDNA synthesis.** LEAP samples are harvested 48h post-transfection of the indicated plasmids and ORF2p-generated cDNA is detected by PCR with a step wise set of ORF2 sequence specific forward primers shown in Figure 7A. Control is LEAP prep on cells transfected with the empty vector (i.e. no ORF2p expression). RNA integrity in LEAP preps and specificity of TRIM28’s effect on ORF2-mediated cDNA generation is assessed with the same set of ORF2 primers and AMV reverse transcriptase as shown in Figure 7A. A PCR product expected to be produced with O4 primer is present in cells expressing ORF2p only and ORF2p co-expressed with either mutant full-length H-TRIM28 (TRIM28 3M) or mutant B box (BB 3M). Targeted sequences are amplified by PCR. O1-O4: amplified sequence of ORF2.

**Supplementary Figure 13. TRIM28 and its B box truncated the synthesized DNA by ORF2p**


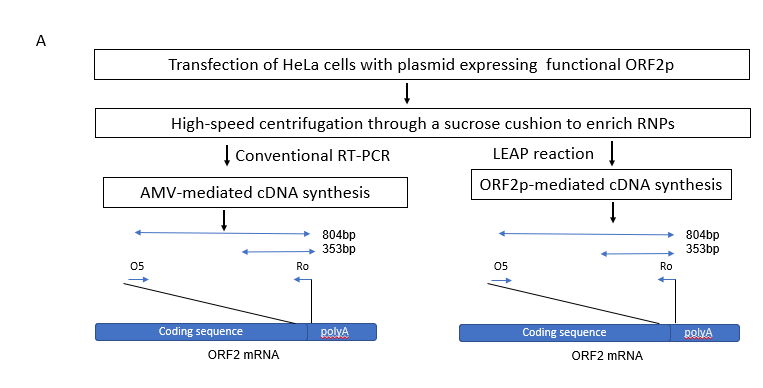


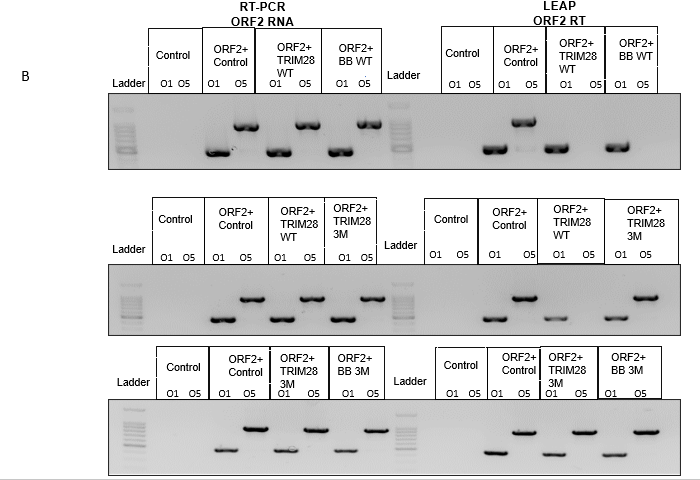


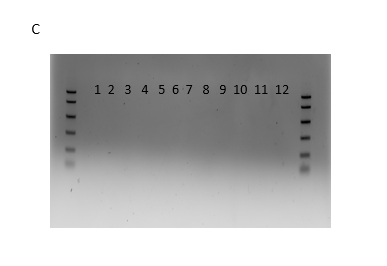


**Supplementary Figure 13. TRIM28 and its B box truncated the synthesized DNA by ORF2p**

**Analysis of cDNA products generated by the ORF2p in HeLa cells transfected with wild-type or mutant TRIM28 or TRIM28 B Box. (A)** Flow chart of the LEAP assay adapted from (2,3). ORF2p-generated cDNA is detected by PCR with the ORF2 sequence specific forward (O5) and reverse (Ro) primers. In parallel, conventional RT-PCR was performed using RNA extracted from HeLa cells transfected with indicated expression plasmids and using the same set of ORF2 primers. Location of primers are shown by horizontal arrows. O5: forward ORF2 specific primer; Ro: reverse primer for ORF2. The expected length of the corresponding PCR products is shown on the right. **(B)** Results of the RT-PCR and LEAP reactions. LEAP samples are prepared by harvesting HeLa cells 48h post-transfection with indicated expression plasmids and analyzed with indicated sets of primers. Control is LEAP prep on HeLa cells transfected with the empty plasmid (i.e. no ORF2p expression). RNA integrity in LEAP preps is assessed with the same set of primers O1 and O5. A PCR product expected to be produced with O5 primer is absent in cells expressing WT full-length H-TRIM28 (TRIM28 WT) or WT B box (BB WT) (Top and middle panels, LEAP). Mutations of three amino acids responsible for multimerization (TRIM28 3M) eliminate this effect (Middle and bottom panels, LEAP). **(C)** Results of the negative control of RT-PCR. LEAP samples are prepared by harvesting HeLa cells 48h post-transfection with indicated expression plasmids and analyzed with O1 set of ORF2 primers in LEAP assay covering a target sequence of 353bp.Distilled water was added instead of AMV RT. Lane 1-12: Control; ORF2+Control; ORF2+TRIM28; ORF2+BB WT; Control; ORF2+Control; ORF2+TRIM28 WT; ORF2+TRIM28 3M; Control; ORF2+Control; ORF2+TRIM28 3M; ORF2+BB 3M.

**Supplementary Figure 14. ORF2p detection in LEAP fractions**


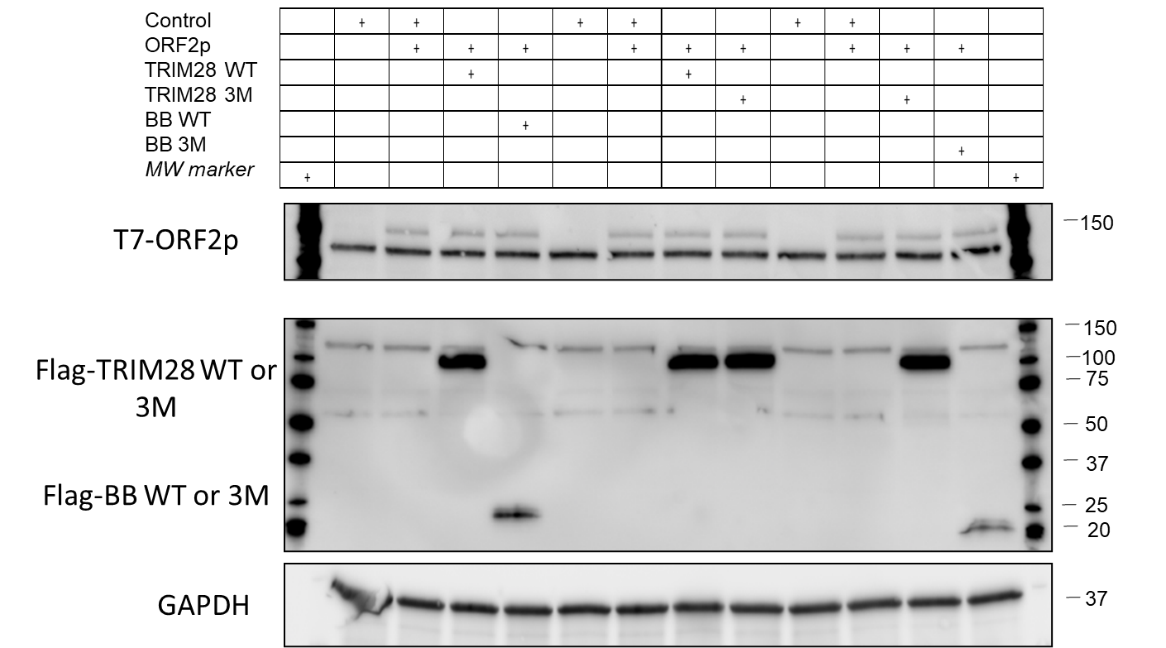


**Supplementary Figure 14. ORF2p detection in LEAP fractions.** LEAP samples were prepared by harvesting HeLa cells 48h post-transfection with indicated expression plasmids. Protein content in these samples was assessed using western blot analysis. ORF2p was detected using anti-T7 antibodies. TRIM28 or BB variants were detected using anti-FLAG antibodies. GAPDH was used as loading control.

**
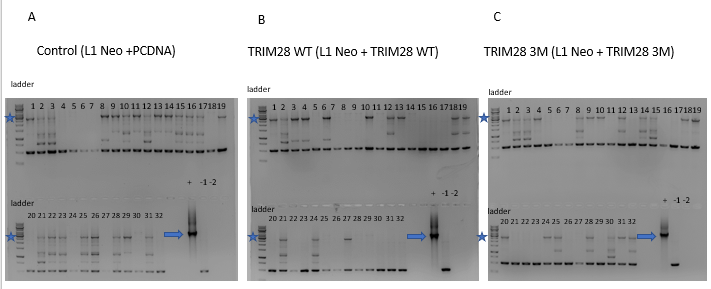
**

**Supplementary Figure 15. Analysis of L1 insertion in genomic DNA using ruler PCR assay**

**Supplementary Figure 15. Analysis of L1 insertion in genomic DNA using ruler PCR assay (33).** PCR on genomic DNA sequence of HeLa cells transfected with L1-neo constructs was performed**.** Three kilobase ruler PCR was performed using primers covering around 3kb of the L1 Neo sequence (33). The L1-neo plasmid gives a band at 3771 bp (indicated by the arrows), while the spliced L1-neo insertion gives a band at 2864 bp (indicated by the stars). Thirty-two clones were randomly picked and subjected to genomic DNA extraction. + indicates the lane with positive control (PCR product from L1-Neo plasmid); -1 lane indicates negative control with PCR product from genomic DNA of un-transfected cells; -2 lane indicates negative control with PCR product using distilled water as template.**(A)** PCR performed on genomic DNA extracted from the G418-resistent clones of HeLa cells co-transfected with L1-Neo expression plasmid and the control plasmid (PCDNA 3.1+ empty vector). **(B)** PCR performed on genomic DNA extracted from the G418-resistant clones of HeLa cells co-transfected with L1-Neo construct and TRIM28 WT expression plasmid. **(C)** PCR performed on genomic DNA extracted from the G418-resistant clones of HeLa cells co-transfected with L1-Neo construct and TRIM28 3M

**Supplementary Figure 16. L1 mobilization in samples collected from patients with endometrial cancer, prostate cancer and ovarian cancer**


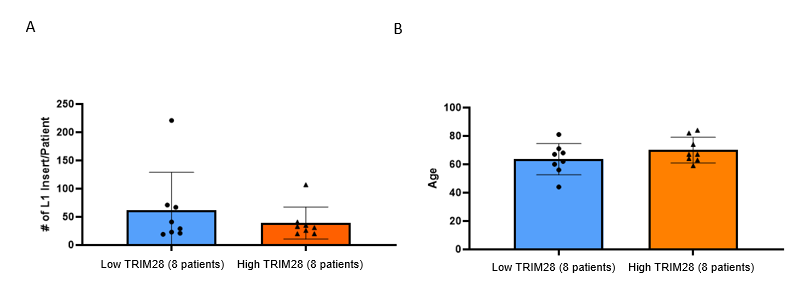


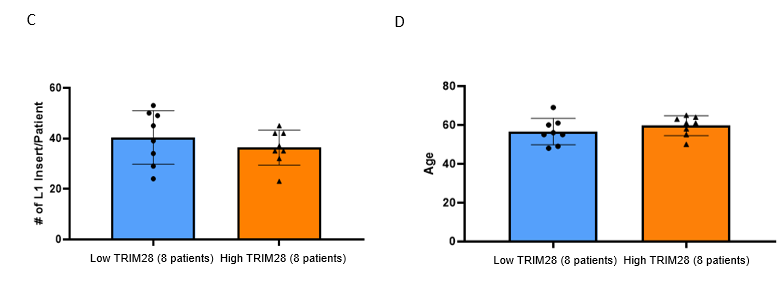


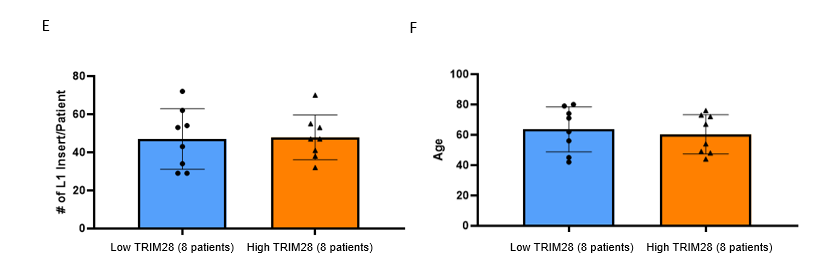


**Supplementary Figure 16. L1 mobilization in samples collected from patients** **with endometrial cancer, prostate cancer, or ovarian cancer.** **(A)** For endometrial cancer patient samples, an average number of the tumor specific L1 inserts per patient as determined by MELT analysis is represented by black dots (Low TRIM28) or triangles (High TRIM28). The average number of inserts per group is not significantly different between the High TRIM28 and Low TRIM28 group of patients (n = 8, t-test, P=0.4). Power analysis of these results determined that a larger sample size (N=142) is needed to detect the difference (power of 0.80 and alpha of 0.05) **(B)** For endometrial cancer patient samples, the average age is not significantly different between the High TRIM28 and Low TRIM28 groups of patients (n = 8, t-test, P=0.22). **(C)** For prostate cancer patients, an average number of the tumor specific L1 inserts per patient as determined by MELT analysis is represented by black dots or triangles. The average number of inserts per group is not significantly different between the High TRIM28 and Low TRIM28 groups of patients (n = 8, t-test, P=0.38). Power analysis of these results determined that a larger sample size (N=160) is needed to detect the difference (power of 0.80 and alpha of 0.05) **(D)** For prostate cancer patients, the average age is not significantly different between the High TRIM28 and Low TRIM28 groups of patients (n = 8, t-test, P=0.3). **(E)** For ovarian cancer patients, an average number of the tumor specific L1 inserts per patient as determined by MELT analysis is represented by black dots (Low TRIM28) or triangles (High TRIM28). The average number of inserts per group is not significantly different between the High TRIM28 and Low TRIM28 groups of patients (n = 8, t-test, P=0.9). Power analysis of these results determined that a larger sample size (N=1570) is needed to detect the difference (power of 0.80 and alpha of 0.05) **(F)** For ovarian cancer patients, the average age is not significantly different between the High TRIM28 and Low TRIM28 group of patients (n = 8, t-test, P=0.6479). For all figures, error bars represent the standard deviation (SD).
